# G-type receptor-like kinase AsNIP43 interacts with rhizobia effector nodulation outer protein P and is required for symbiosis

**DOI:** 10.1093/plphys/kiad318

**Published:** 2023-07-11

**Authors:** Yuan Liu, Ye Lin, Feng Wei, Yanfei Lv, Fuli Xie, Dasong Chen, Hui Lin, Youguo Li

**Affiliations:** State Key Laboratory of Agricultural Microbiology, College of Life Science and Technology, Huazhong Agricultural University, Wuhan 430070, Hubei Province, China; State Key Laboratory of Agricultural Microbiology, College of Life Science and Technology, Huazhong Agricultural University, Wuhan 430070, Hubei Province, China; State Key Laboratory of Agricultural Microbiology, College of Life Science and Technology, Huazhong Agricultural University, Wuhan 430070, Hubei Province, China; State Key Laboratory of Agricultural Microbiology, College of Life Science and Technology, Huazhong Agricultural University, Wuhan 430070, Hubei Province, China; State Key Laboratory of Agricultural Microbiology, College of Life Science and Technology, Huazhong Agricultural University, Wuhan 430070, Hubei Province, China; State Key Laboratory of Agricultural Microbiology, College of Life Science and Technology, Huazhong Agricultural University, Wuhan 430070, Hubei Province, China; State Key Laboratory of Agricultural Microbiology, College of Life Science and Technology, Huazhong Agricultural University, Wuhan 430070, Hubei Province, China; State Key Laboratory of Agricultural Microbiology, College of Life Science and Technology, Huazhong Agricultural University, Wuhan 430070, Hubei Province, China

## Abstract

In the Rhizobium-Legume symbiosis, the nodulation outer protein P (NopP) effector is one of the key regulators for rhizobial infection and nodule organogenesis. However, the molecular mechanism through which host legume plants sense NopP remains largely unknown. Here, we constructed an *nopP* deletion mutant of *Mesorhizobium huakuii* and found that *nopP* negatively regulates nodulation on Chinese milk vetch (*Astragalus sinicus*). Screening for NopP interacting proteins in host plants using the yeast 2-hybrid system identified NopP interacting protein 43 (AsNIP43), which encodes a G-type receptor-like kinase (LecRLK). The B-lectin domain at the N terminus of AsNIP43 was essential in mediating its interaction with NopP, which was confirmed in vitro and in vivo. Subcellular localization, co-localization, and gene expression analyses showed that AsNIP43 and NopP function tightly associated with earlier infection events. RNA interference (RNAi) knockdown of *AsNIP43* expression by hairy root transformation led to decreased nodule formation. AsNIP43 plays a positive role in symbiosis, which was further verified in the model legume *Medicago truncatula*. Transcriptome analysis indicated that *MtRLK* (a homolog of *AsNIP43* in *M. truncatula*) may function to affect defense gene expression and thus to regulate early nodulation. Taken together, we show that LecRLK AsNIP43 is a legume host target that interacts with rhizobia effector NopP is essential for rhizobial infection and nodulation.

## Teaser

A legume host target G-type lectin receptor-like kinase interacts with rhizobia T3SS effector nodulation outer protein P and is essential for rhizobial infection and nodulation.

## Introduction

The occurrence, development, and nitrogen-fixing function of legume nodules are attributed to the mutual recognition and exchange of signal molecules and specific determinants between microorganisms and the host plants. The flavonoid compounds secreted by legume roots can activate a set of nodulation genes from rhizobia to produce Nod factors (NFs), a signal molecule of lipo-chitooligosaccharides (Oldroyd et al. 2011; Ferguson et al. 2019). Subsequently, the NFs are perceived by the LysM-receptor-like kinase of host legumes, which causes the polar growth of root hairs, cell membrane invagination, and endothelial cell division, resulting in infection thread formation and root nodule organogenesis (Madsen et al. 2010; Oldroyd 2013). In addition, rhizobia possess various carbohydrates on their surface, such as lipopolysaccharides, exopolysaccharides (EPS), capsular polysaccharides, and cyclic glucans. Polysaccharides can determine symbiotic host-specificity (Perret et al. 2000). Another LysM-type receptor-like kinase EPR3 (exopolysaccharide receptor 3) can recognize the EPS of rhizobia and serve as the receptor during the infection process (Kawaharada et al. 2015, 2017). The Type III effectors, also known as nodulation outer proteins (Nops) and secreted via the rhizobial Type III secretion system (T3SS), also play an important role in host-specific symbiosis (Shamseldin 2022).

The T3SS has been extensively studied in plant pathogens, which delivers the effectors directly into the eukaryotic host cells to suppress plant immunity (PTI) activated by microbial inducer (pathogen-associated molecular pattern, PAMP) and facilitates the infection (Cao et al. 2017). Similar to pathogens, some rhizobia also possess T3SS and secrete Nops during the infection process in symbiosis with legume (Miwa and Okazaki 2017). Rhizobial T3SS may have different effects depending on the host legumes (Yasuda et al. 2016). On the one hand, the effectors can promote symbiosis by evading the host immune response; on the other hand, they are also recognized by the intracellular resistance (R) proteins in legume plants and trigger strong defense responses to cause hypersensitive cell death, which is called effector-triggered immunity (ETI). ETI will result in a prevention of infection and symbiotic incompatibility (Yang et al. 2010; Sugawara et al. 2018). Interestingly, the T3SS effector can sometimes trigger symbiosis in some way independent of the NF signaling pathway. For example, both wild-type *Bradyrhizobium elkanii* and NF-deficient *B. elkanii* can induce nitrogen-fixing nodules with soybean (*Glycine max*) *nfr* mutant (Okazaki et al. 2013).

Mutation analysis has indicated that the T3SS effector plays a vital role in symbiosis (Okazaki et al. 2016). ErnA (effector required for nodulation-A) is a Nop effector of *Bradyrhizobium* sp. ORS3257, which can contribute to nodulation with *Aeschynomene indica* independent of NFs (Teulet et al. 2019). The NopL effector of *Rhizobium* sp. NGR234 is multiply phosphorylated by MAP kinases and interferes with the MAP kinase signaling, thereby preventing the senescence of premature nodules on *Phaseolus vulgaris* (Zhang et al. 2011; Ge et al. 2016). NopM of *Rhizobium* sp. NGR234 is an E3 ubiquitin ligase, which promotes symbiotic initiation with *Lablab purpureus* by reducing the ROS level (Xin et al. 2012). NopD of *Bradyrhizobium* sp. XS1150 possesses the SUMO protease activity and plays a negative regulatory role in symbiosis with *Tephrosia vogelii* (Xiang et al. 2020). However, a rhizobia-specific effector NopP does not contain any known functional domains and has no homology with any avirulence effectors in pathogens (Ausmees et al. 2004). NopP can positively or negatively regulate nodulation depending on the host plant. NopP of *Rhizobium* sp. NGR234 can be phosphorylated by legume root kinases, and its mutation led to a decrease in root nodule number on *Flemingia congesta* and *T. vogelii* (Skorpil et al. 2005). The NopP of *Bradyrhizobium diazoefficiens* USDA122 is the determinant of symbiotic incompatibility with *Rj2*-soybean, and the R proteins associated with specific NopP variants can trigger the ETI to control rhizobial infection (Yang et al. 2010; Sugawara et al. 2018). It has reported that a recently identified R protein GmNNL1 directly interacts with the effector NopP of *B. diazoefficiens* USDA110, triggering ETI of the host, preventing rhizobial invasion through root hairs, and inhibiting symbiotic nodulation (Zhang et al. 2021). And another target of *Mesorhizobium amorphae* NopP, TRAPPC13, has been identified recently, the effector and its target play a role during the early infection process (Liu et al. 2021). However, the targets and functions of NopP in other legumes-rhizobia symbiosis have not been fully studied.

*Astragalus sinicus* is a traditional green manure legume which is widely cultivated in southern China, Japan, and Korea. It can be used to enhance the fertility of rice fields, and can also be used as pasture and nectar plants (Cho et al. 1995). *A. sinicus* can establish symbiosis with *Mesorhizobium huakuii*, forming indeterminate nodules. *M. huakuii* 7653R was isolated from *A. sinicus* root nodules, and the symbiosis system has been studied and applied extensively (Lei et al. 2014; Zhou et al. 2019; Si et al. 2020). The strain 7653R contains a single copy of T3SS-secreted effector NopP (Wang et al. 2014). However, the role of NopP in the symbiosis between *A. sinicus* and *M. huakuii* 7653R remains unknown. In this study, we characterized the NopP of *M. huakuii* 7653R and obtained the interacting receptor-like kinase AsNIP43 (accession no. MT435087) in legume *A. sinicus* by a yeast 2-hybrid system. The interaction was confirmed by in vivo and in vitro experiments. Symbiotic phenotypes showed that AsNIP43 plays a vital role in rhizobial infection, which was verified in the model legume *Medicago truncatula*. Taken together, our findings identified a legume LecRLK AsNIP43, a legume host target that interacts with rhizobia effector NopP, and also revealed that legume lectin RLK plays an essential role in the establishment of symbiotic infection and nodulation.

## Results

### *Mesorhizobium* NopP participated in rhizobial infection and negatively regulated root nodule number on *A. sinicus*

In the genome of *M. huakuii* 7653R, its symbiotic plasmid b (pMHb) possesses a complete T3SS gene cluster, and it is induced by flavonoids. There is a *tts* box upstream *nopP*. NopP is an effector secreted by the T3SS of 7653R (Supplemental Fig. S1). The 7653R NopP (QGU20929.1), which consisted of 281 amino acid residues, with a calculated molecular weight of 31.3 kDa. Bioinformatics analysis by EffectiveDB showed that NopP had an N-terminal secretory signal peptide but without any conserved functional domain, showing no homology to the effectors in plant pathogens (Eichinger et al. 2016). Phylogenetic analysis revealed that rhizobial NopPs were distributed in 3 main branches corresponding to *Sinorhizobium*, *Mesorhizobium*, and *Bradyrhizobium* (Supplemental Fig. S2), indicating that NopP may play diverse and differential roles in different types of rhizobia-legume symbiosis.

**Figure 1.**
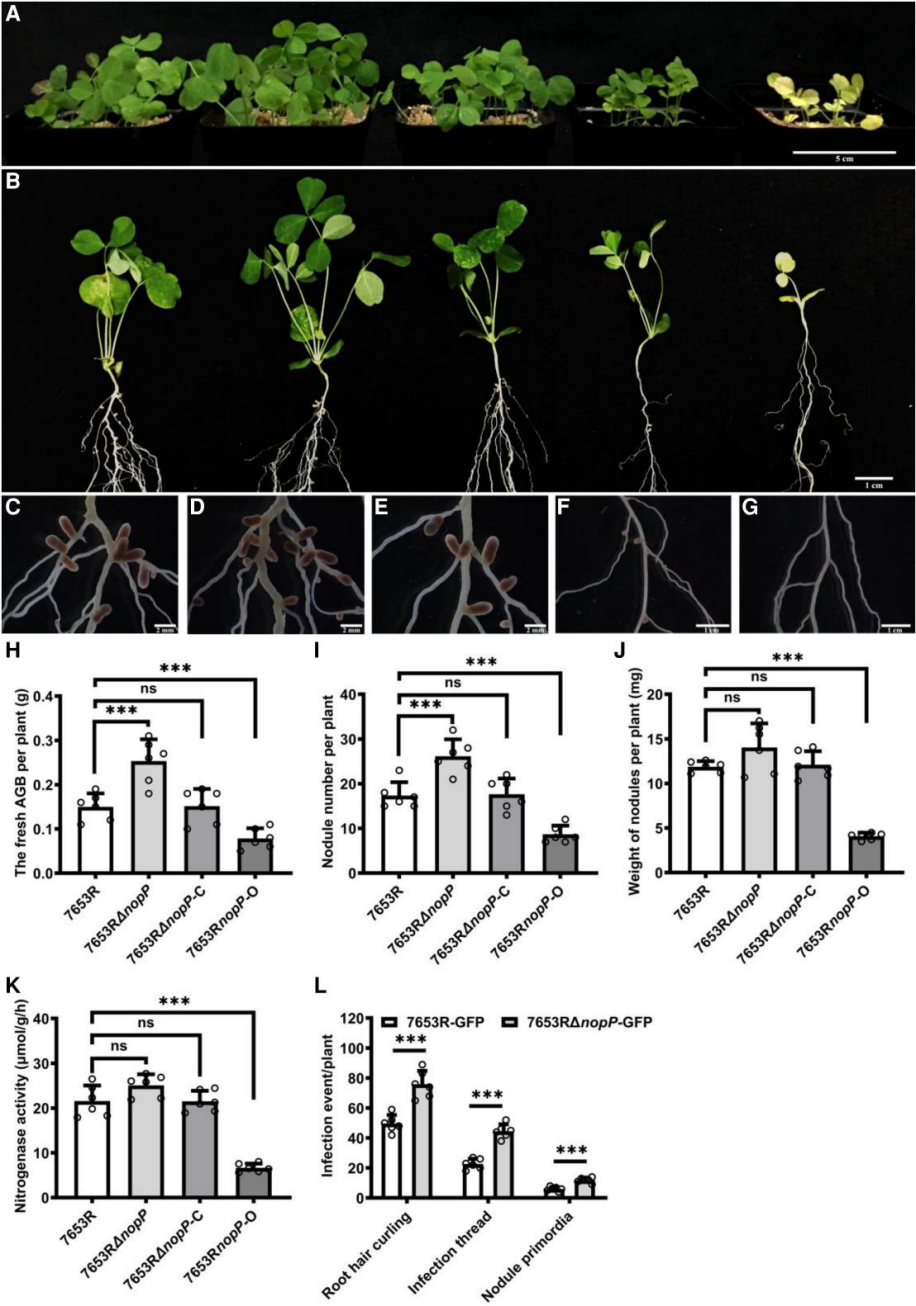
Symbiotic phenotypes of *M. huakuii* 7653RΔ*nopP* on the host plant *A. sinicus*. Plants were inoculated with indicated strains and harvested at 28 dpi. **A to G)** From left to right, the plants were inoculated with 7653R, 7653RΔ*nopP*, 7653RΔ*nopP*-C, 7653R*nopP*-O, and without inoculation. **A)** Aerial part phenotypes; **B)** whole-plant phenotypes; **C to G**) root nodule phenotypes; **H** to **K)** fresh aboveground biomass (AGB), nodule numbers, nodule weights, and the nitrogenase activity of plants inoculated with 7653R, 7653RΔ*nopP*, 7653RΔ*nopP*-C, and 7653R*nopP*-O. **L)** Infection events of GFP-labeled 7653R and 7653RΔ*nopP* at 7 dpi (*n* = 6). Scale bars = 5 cm **A**), 1 cm **B**), 2 mm **C to E**) and 1 cm **F and G**). Significance: ***P < 0.001; **P < 0.01; *P < 0.05; ns, not significant; Student's *t*-test. Data are shown as mean ± Sd. The error bars represent the Sds of 3 independent experiments.

To investigate the function of *nopP* in symbiosis, a *nopP* deletion mutant of *M. huakuii* 7653R (named as 7653RΔ*nopP*) was constructed (bacterial strains and plasmids in Supplemental Table S1). For the functional complementary strain 7653RΔ*nopP*-C, in which a recombinant plasmid pBBR1MCS-5-pro*nopP* + *nopP* was introduced into the 7653RΔ*nopP* mutant, while the overexpression strain 7653R*nopP*-O, a plasmid pBBR1MCS-5-*nopP* was introduced into wild-type 7653R (primers in Supplemental Table S2). Growth assay under free-living conditions demonstrated that the deletion of *nopP* did not affect the free-living growth of 7653RΔ*nopP* (Supplemental Fig. S3). We examined the transcription levels of *nopP* in mutants under free-living condition and found that there is no significant difference in the expression level of *nopP* between the complementary strain and wild-type 7653R strain, while the expression level of *nopP* in the overexpression strain is over 70 times higher than that of in wild-type strains (Supplemental Fig. S4). Examination of the symbiotic phenotypes revealed that the mutant 7653RΔ*nopP* induced significant increases in nodule number and above-ground biomass per plant on *A. sinicus*. The strain 7653RΔ*nopP*-C resulted in a similar symbiotic phenotype on *A. sinicus* as the wild-type strain, indicating that the *nopP* mutant phenotype could be functionally restored. The strain 7653R*nopP*-O induced significant reductions of nodules and shoot biomass per plant relative to the WT strain (Fig. 1). To investigate the effect of 7653RΔ*nopP* on the symbiotic infection, we labeled the mutant with GFP and obtained a strain named as 7653RΔ*nopP*-GFP with constitutive GFP expression. The early infection events of wild-type *A. sinicus* inoculated with 7653RΔ*nopP*-GFP and 7653R-GFP, including root hair curling, infection threads, and nodule primordia were observed and quantified at 3, 5, 7 d post inoculation (dpi), separately. It was found that the mutant formed significantly more root hair curling, infection threads, and nodule primordia (Fig. 1L), which was consistent with the increase in nodule number.

Considering a global gene expression profile and changes may provide molecular insights into the understanding on the symbiotic phenotype of 7653RΔ*nopP*, we conducted a transcriptome sequencing analysis for the roots inoculated with wild-type 7653R and 7653RΔ*nopP*, respectively. The roots inoculated with wild-type 7653R were used as the control. According to RNA sequencing (RNA-seq) results (Supplemental Data Set 1), we at first analyzed the differentially expressed genes (DEGs) and performed KEGG enrichment analysis on the DEGs in the 2 groups. At 1 dpi, the KEGG pathway of DEGs was in enriched in MAPK signaling pathway-plant, plant hormone signal transduction, phenylpropanoid biosynthesis, isoflavonoid biosynthesis, and other signaling pathways; and at 6 dpi, the KEGG pathway of DEGs was in enriched in plant–pathogen interaction, MAPK signaling pathway-plant, plant hormone signal transduction, flavone and flavonol biosynthesis, nitrogen metabolism, and other signaling pathways (Supplemental Fig. S5). The results show that the DEGs are mainly involved in signaling pathways such as bacterial–plant interaction, signal transduction, flavonoid biosynthesis, and nitrogen metabolism. All of them are closely related to the symbiotic interactions and nodulation. Besides, NopP is a determinant of symbiotic specificity and is generally thought to trigger defense responses in symbiotic-incompatible systems (Sugawara et al. 2018). Therefore, we further had a closer check on the differential expression of defense response-related genes in the host. It found that, in the 7653RΔ*nopP* roots at 1 and 6 dpi, the genes related to defense response showed downregulated expression. The proteins encoded by these genes included WRKY transcription factor, NBS-LRR class protein, disease resistance/-like protein, defensin-like protein, and pathogenesis-related protein (Tables 1 and 2). It implies that 7653RΔ*nopP* inoculation may result in a weakened defense response in the host plant, and therefore promoted more root nodules formation.

**Table 1.** DEGs related to defense response in WT-1 vs Δ*nopP*-1 group

ID	log2FC	Regulated	Description
c47949.graph_c0	−1.06034	Down	Defense mechanisms
c50370.graph_c0	−1.57819	Down	TMV resistance protein
c47690.graph_c0	−0.75217	Down	WRKY transcription factor
c57850.graph_c1	−0.84358	Down	Disease resistance-like protein
c59369.graph_c0	−1.87521	Down	Defense mechanisms
c52125.graph_c0	−0.72344	Down	Defense mechanisms
c59862.graph_c0	−0.86967	Down	NBS-LRR class protein
c59818.graph_c1	−0.7792	Down	Disease resistance-like protein
c57189.graph_c1	−0.83279	Down	Defense mechanisms
c60055.graph_c0	−0.94559	Down	Disease resistance protein
c50393.graph_c1	−2.1491	Down	Defense mechanisms
c56492.graph_c1	−0.69807	Down	Defense mechanisms
c58198.graph_c1	−0.77064	Down	Disease resistance-like protein

**Table 2.** DEGs related to defense response in WT-6 vs Δ*nopP*-6 group

ID	log2FC	Regulated	Description
c58235.graph_c0	−1.33661	Down	Disease resistance protein
c60046.graph_c0	−0.88461	Down	TMV resistance protein
c60090.graph_c0	−0.8476	Down	TMV resistance protein
c46033.graph_c1	−1.37052	Down	WRKY transcription factor
c38556.graph_c0	−2.61058	Down	Defensin-like protein
c50222.graph_c0	−0.93124	Down	WRKY transcription factor
c51013.graph_c1	−0.95952	Down	Disease resistance protein
c48902.graph_c0	−1.0066	Down	Disease resistance protein
c46033.graph_c0	−1.5175	Down	WRKY transcription factor
c59471.graph_c0	−1.87474	Down	Disease resistance protein
c46766.graph_c0	−0.80677	Down	WRKY transcription factor
c58419.graph_c0	−0.794	Down	Disease resistance protein
c51013.graph_c3	−0.9102	Down	Disease resistance protein
c44284.graph_c0	−1.44361	Down	TMV resistance protein
c57670.graph_c0	−1.07539	Down	Disease resistance protein
c44439.graph_c0	−2.76374	Down	Disease resistance protein
c60055.graph_c0	−0.99258	Down	Disease resistance protein
c51013.graph_c2	−1.03758	Down	Disease resistance protein
c46157.graph_c0	−1.17348	Down	Pathogenesis-related protein
c50222.graph_c1	−0.82846	Down	WRKY transcription factor
c49004.graph_c0	−1.24132	Down	Disease resistance protein
c56159.graph-c1	−0.64047	Down	Disease resistance protein
c42123.graph_c0	−2.99212	Down	Defensin-like protein
c53663.graph_c1	−0.98595	Down	Disease resistance protein

### A legume G-type receptor-like kinase interacted with the *Mesorhizobium* T3SS effector NopP

**Figure 2.**
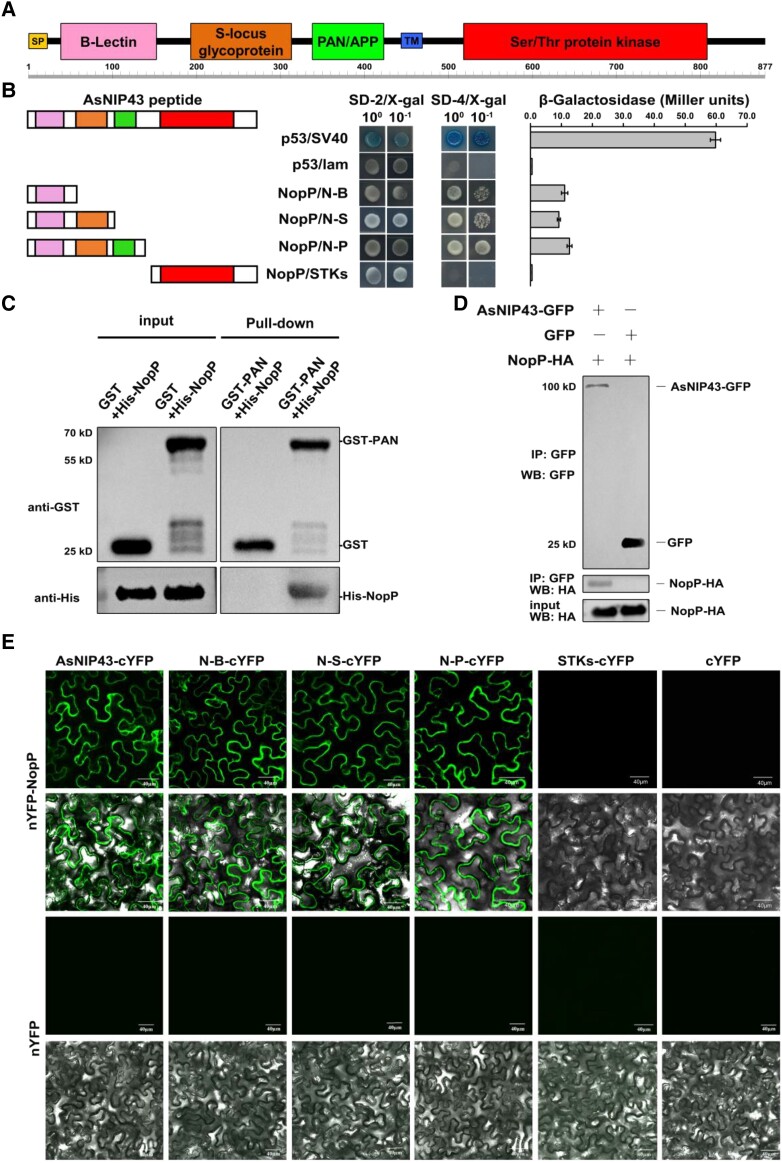
Interaction between AsNIP43 and NopP. **A)** AsNIP43 protein scheme showing predicted conserved domains. SP, signal peptide; TM, transmembrane; B-lectin, Bulb-type mannose-specific lectin; PAN/APP, plasminogen-apple-nematode-like domain. **B)** Yeast 2-hybrid assay on the interaction between AsNIP43 truncated constructs and NopP. The strength of interaction was evaluated by detecting the β-galactosidase activities. Data are mean ± Sd. N-B, B-lectin domain; N-S, B-lectin and S-locus glycoprotein domains; N-P, B-lectin, S-locus glycoprotein and PAN/APP domains, also named as PAN. **C)** In vitro protein pull-down assay for the interaction of AsNIP43 and NopP. His-NopP was pulled down by GST-PAN. **D)** CoIP assay for AsNIP43 and NopP. HA-NopP was immunoprecipitated by AsNIP43-GFP. **E)** BiFC of the interaction between AsNIP43 truncated constructs and NopP in planta. Strong fluorescence signals were observed between AsNIP43, N-B, N-S, N-P (PAN), and NopP. No fluorescence signals were detected between STKs and NopP and the negative controls. Scale bar, 40 *μ*m.

To identify the NopP-interacting proteins in the host plant, the NopP of *M. huakuii* 7653R was used as a bait to screen the *A. sinicus* complementary DNA (cDNA) yeast 2-hybrid library. Firstly, we examined that the bait pGBKT7-NopP has no self-activation activity and no toxicity affecting the growth of yeast, which can be used for library screening. As a result, 12 candidate interaction proteins were obtained by sequencing (Supplemental Table S3). Sequence alignment indicated that one of them was a G-type lectin receptor-like kinase (LecRLK), which was designated as AsNIP43. We obtained the full length of *AsNIP43* through rapid amplification of cDNA ends method, including the coding region and untranslated region (UTR), and verified the coding region sequence through our transcriptome data. *AsNIP43* contains one exon and does not contain any introns. Bioinformatics analysis showed that *AsNIP43* had an ORF of 2,634 bp and encoded 877 amino acid residues. The AsNIP43 protein was a G-type receptor-like kinase, containing a typical signal peptide, B-lectin, S-locus, and plasminogen/apple/nematode (PAN)-APP domains at the N terminus, and a conserved serine/threonine kinase domain at the C terminus. Besides, it had a single-pass transmembrane segment (Fig. 2A). The deduced amino acid sequences of these domains were conserved among legume homologous proteins (Supplemental Fig. S6). Phylogenetic analysis revealed that AsNIP43 homologs exist in both legume plants and nonlegume plants (Supplemental Fig. S7).

AsNIP43 contained an ectodomain and intracellular domain. To identify the essential domain for the interaction with NopP, we constructed a series of truncated AsNIP43-AD fusion expression vectors and performed self-activation verification. The yeast 2-hybrid experiment and β-galactosidase activity measurement showed that 3 groups of truncation experiments including B-lectin, B-lectin + S-locus, B-lectin + S-locus + PAN-APP domains all interacted with NopP. This indicates that B-lectin domain is sufficient to mediate the interaction of NopP-AsNIP43 (Fig. 2B).

Pull-down, co-immunoprecipitation (CoIP) and bimolecular fluorescence complementation (BiFC) assays were carried out to further confirm the interaction between AsNIP43 and NopP. As shown in Fig. 2C, His-NopP protein was pulled down by the GST-PAN resin instead of by the GST protein or the resin, indicating protein–protein interaction between AsNIP43 and NopP in vitro. For the Co-IP assay, NopP was co-immunoprecipitated with AsNIP43-GFP but not with GFP (Fig. 2D), indicating the interaction of AsNIP43 with NopP in vivo.

Further, we validated their interaction by BiFC. As a result, fluorescence signals were observed in groups co-expressing nYFP-NopP and cYFP-AsNIP43 or nYFP-NopP and cYFP-ectodomain, while no fluorescence signals were detected in the group co-expressing nYFP-NopP and cYFP-STKs or the negative control (Fig. 2E). Fluorescence signals were mainly localized in the cytoplasmic membrane of epidermal cells. The above experimental results confirmed that AsNIP43 interacts with NopP in planta.

### AsNIP43 co-localized with NopP in planta

**Figure 3.**
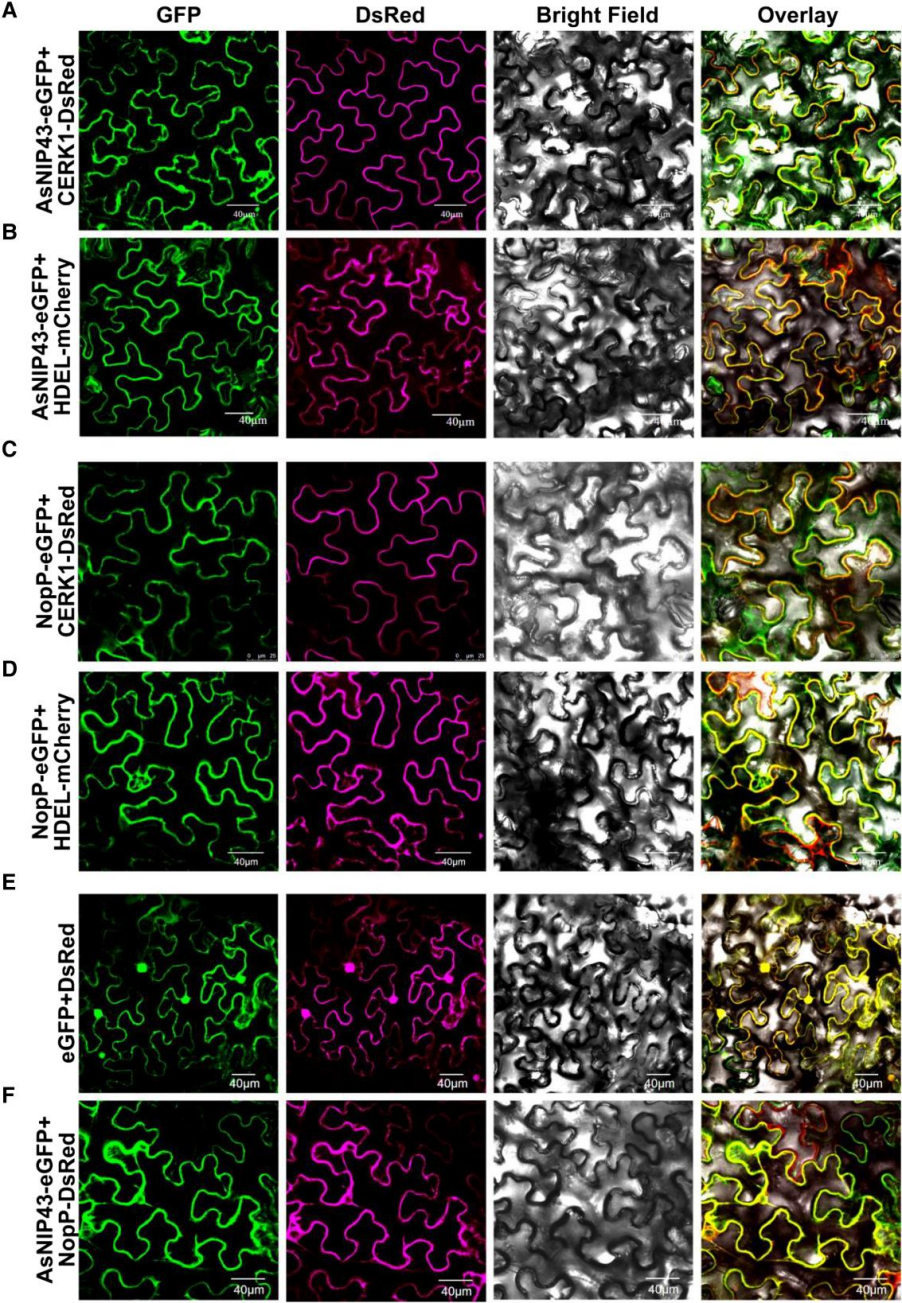
Subcellular location and colocalization of AsNIP43 and NopP in *N. benthamiana* cells. **A** and **B)** Confocal microscopy images of the *N. benthamiana* leaf cells co-expressing AsNIP43-GFP with the plasma membrane marker CERK1-DsRed, and the ER marker HDEL-mCherry. **C** and **D)** Confocal microscopy images of *N. benthamiana* cells co-expressing NopP-GFP with the plasma membrane marker, or the ER marker. **E)** Localization of free eGFP and DsRed from control vectors. **F)** Co-localization of AsNIP43-GFP and NopP-DsRed. From left to right, GFP channel, DsRed channel, bright field channel, and overlay. Scale bars, 40 *μ*m **A, B, D to F**), 25 *μ*m **C**).

The subcellular localization of proteins can usually provide useful information for understanding their biological functions. Therefore, the subcellular localization of AsNIP43 and NopP in *Nicotiana benthamiana* epidermal leaf cells was investigated. Enhanced green fluorescent protein (eGFP) was fused to the C terminus of AsNIP43 and NopP driven by the cauliflower mosaic virus 35S promoter. The markers reported to specifically localized to the plasma membrane and endoplasmic reticulum (ER) were used as the control (Miya et al. 2007; Batistic et al. 2010). As expected, the markers CERK1-DsRed and HDEL-mCherry were localized at the plasma membrane and ER membrane, respectively. As shown in Fig. 3, A to D, both AsNIP43-GFP and NopP-GFP were co-localized with the plasma membrane marker and the ER marker. The prerequisite for the interaction between proteins is that they have the same subcellular localization. GFP and DsRed, GFP-tagged AsNIP43, and DsRed-tagged NopP were co-expressed in the *N. benthamiana* leaf cells, respectively. The results showed that GFP and DsRed were co-localized throughout the leaf cells (Fig. 3E), and AsNIP43-GFP was co-localized with NopP-DsRed at the plasma membrane and ER (Fig. 3F).

### Expression patterns of *nopP* and *AsNIP43* during nodulation

In order to explore the functional relevance of AsNIP43 and NopP, we studied the expression patterns of *AsNIP43* and *nopP* in *A. sinicus* noninoculated roots, roots and nodules inoculated with *M. huakuii* 7653R. We used the promoters of *AsNIP43* and *nopP* to drive the expression of β-glucuronidase reporter (GUS).

**Figure 4.**
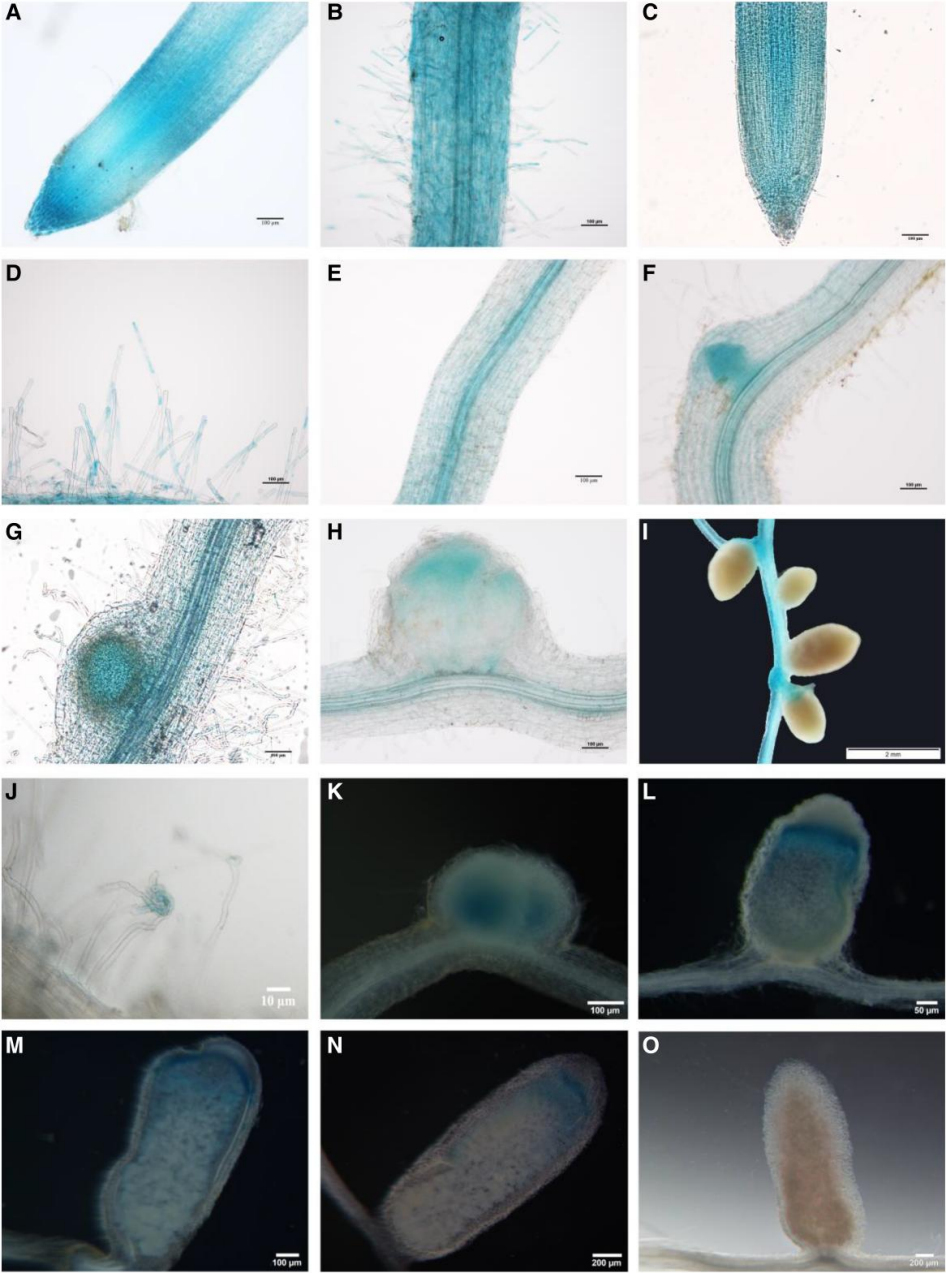
In situ expression pattern of *AsNIP43* and *nopP* during infection and nodulation. Analysis of the expression pattern of *AsNIP43* by GUS staining. **A** and **B)** In the absence of *M. huakuii* 7653R, *AsNIP43* was highly expressed in root epidermal cells, root tips, and root hairs. **C to I)** After rhizobium inoculation, *AsNIP43* was expressed in root tips **C)**, root hairs **D)**, vascular tissues **E)**, lateral root primordia **F)**, nodule primordia **G)**, and infection zone of developing nodules **H)**, but not in mature nodules **I)**. **J to O)** Histochemical GUS staining of root hairs and nodules induced by *M. huakuii* 7653R carrying recombinant plasmid *nopP*_Pro_-GUS. The *A. sinicus* plant roots and nodules were harvested at 3 **J)**, 6 **K)**, 13 **L)**, 18 **M)**, 24 **N),** and 30 **O)** dpi. Scale bars, 10 *μ*m **J)**, 50 *μ*m **L),** 100 *μ*m **A to H, K, M**), 200 *μ*m **N and O**), and 2 mm **I**).

Firstly, we examined the expression pattern of *AsNIP43*. The promoter of *AsNIP43* was cloned via inverse PCR and used to drive the expression of GUS reporter. Expression of the *pAsNIP43*:GUS construct was then monitored in *A. sinicus* transformed hairy roots. The results showed that in both noninoculated roots and inoculated roots, the expression of *pAsNIP43*:GUS was observed in the epidermal cells including root hairs (Fig. 4, A to D). It was also expressed in the cortical cells of inoculated roots and the lateral root base (Fig. 4, C, E and F). Notably, *AsNIP43* had high expression in nodule primordia and the infection zone of young nodules, but no signals were detected in mature nodules (Fig. 4, G to I). These results were consistent with the symbiotic expression patterns monitored by RT-qPCR (Supplemental Fig. S8A). All of these results indicated that AsNIP43 may function at the early stage of rhizobial infection rather than the stage of nodule maturation.

In addition, we investigated the spatial expression pattern of *nopP* during symbiosis. A 600-bp promoter of *nopP*, the genomic DNA fragment upstream of the start codon containing the full promoter sequence, was cloned and fused with a GUS reporter. The *pnopP*:GUS construct was introduced into *M. huakuii* 7653R, which was subsequently used to inoculate *A. sinicus* seedlings. At 3 dpi, GUS activity was detected at root hair curling (Fig. 4J). Subsequently, GUS signals were observed in young nodules (Fig. 4K), particularly in the infection zone (Fig. 4, L to N), but not in mature nodules (Fig. 4O). The GUS activity results were also consistent with the RT-qPCR results (Supplemental Fig. S8B), suggesting that *nopP* may be involved in the rhizobial infection during symbiosis. The similar expression patterns of *AsNIP43* and *nopP* implied that they might function together during the infection process of symbiosis.

### *AsNIP43* knockdown impaired rhizobial infection and nodulation while *AsNIP43* overexpression increased nodule number

**Figure 5.**
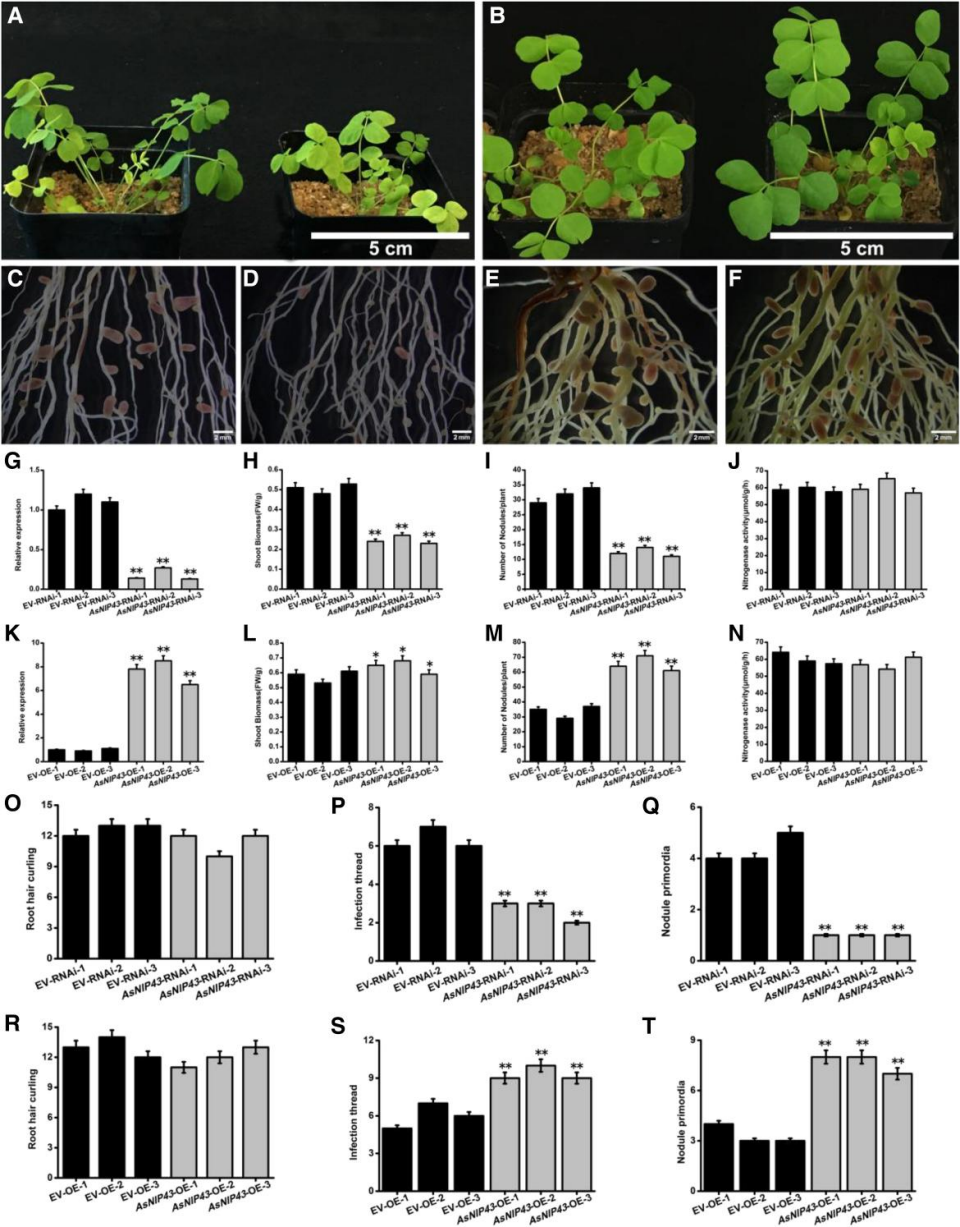
Symbiotic phenotypes induced by *M. huakuii* 7653R on *A. sinicus AsNIP43-*RNAi lines and plants overexpressing *AsNIP43*. **A)** Overall growth of the EV-RNAi line and *AsNIP43-*RNAi line of *A. sinicus*. **B)** Overall growth of the EV-OE line and *AsNIP43-*OE line of *A. sinicus*. **C)** Root and nodule phenotypes of the EV-RNAi line of *A. sinicus* hairy roots inoculated with *M. huakuii* 7653R. **D)** Root and nodule phenotypes of the *AsNIP43-*RNAi line of *A. sinicus* hairy roots inoculated with *M. huakuii* 7653R. **E)** Root and nodule phenotypes of the EV-OE line of *A. sinicus* hairy roots inoculated with *M. huakuii* 7653R. **F)** Root and nodule phenotypes of the *AsNIP43*-OE line of *A. sinicus* hairy roots inoculated with *M. huakuii* 7653R. **G)** Transcript levels of *AsNIP43* in EV-RNAi lines and *AsNIP43-*RNAi lines detected by real-time RT-qPCR. **H to J**) Shoot biomass, nodule number, and nitrogenase activity of EV-RNAi lines and *AsNIP43-*RNAi lines. **K)** Transcript levels of *AsNIP43* in EV-OE lines and *AsNIP43-*OE lines detected by RT-qPCR analysis. **L to N**) Shoot biomass, nodule numbers, and the nitrogenase activity of EV-OE lines and *AsNIP43-*OE lines. **O to Q)** Frequencies of infection events per hairy root of EV-RNAi lines and *AsNIP43*-RNAi lines. **R to T**) Frequencies of infection events per hairy root of EV-OE lines and *AsNIP43*-OE lines. Significances: **P < 0.01; *P < 0.05; Student's *t*-test. Each column represents an independent transgenic plant line. Scale bars, 5 cm **A and B**), 2 mm **C to F**). The error bars represent the Sds of 3 independent experiments.

To investigate the symbiotic phenotype and function of *AsNIP43* in the nodulation process, RNAi and overexpression experiments of *AsNIP43* were performed. In order to avoid off-target silencing, the fragment specifically silencing the expression of the *AsNIP43* was used to construct the RNAi vector. Eighteen independent RNAi lines were generated for the analysis of symbiotic phenotypes. We observed the differences in symbiotic phenotypes of the *AsNIP43-*RNAi lines and empty-vector control plants inoculated with *M. huakuii* 7653R (Fig. 5, A, C and D). And the silencing of *AsNIP43* was confirmed by RT-qPCR (Fig. 5G). The silencing of *AsNIP43* resulted in significant decreases in nodule number and shoot biomass per plant compared with the control plants (Fig. 5, H and I). However, the RNAi-nodules demonstrated similar color of light pink and nitrogenase activity per gram of nodule compared with the controls (Fig. 5J). These results indicated that *AsNIP43* is involved in regulating nodule number rather than nodule development in *A. sinicus*.

To further investigate the role of *AsNIP43* in symbiosis, we generated transgenic *A. sinicus* roots overexpressing *AsNIP43* under the control of 35S promoter. A total of 23 independent overexpression lines (*AsNIP43-*OE lines) were obtained and analyzed for symbiotic phenotypes. We observed the differences in symbiotic phenotypes of the *AsNIP43-*OE lines and empty-vector control plants inoculated with *M. huakuii* 7653R (Fig. 5, B, E, and F). The expression level of *AsNIP43* in transgenic plants was confirmed by RT-qPCR (Fig. 5K). The *AsNIP43-*OE lines exhibited a remarkable increase in nodule number but no difference in shoot biomass relative to the control (Fig. 5, L and M). The nitrogenase activity per gram of OE-nodules was no different from the controls (Fig. 5N). These results further confirmed the function of *AsNIP43* in the regulation of nodule number in *A. sinicus*-*M. huakuii* 7653R symbiosis.

To investigate which step of the rhizobial infection was affected in the *AsNIP43-*RNAi and *AsNIP43-*OE lines during nodulation, the early infection events were observed and quantified. All the transgenic plants inoculated with *M. huakuii* 7653R labeled with GFP. The *AsNIP43-*OE and RNAi plants showed no significant difference from the control in the number of root hair curling (Fig. 5, O and R). The number of infection threads and primordia was significantly increased in OE lines while significantly reduced in the RNAi lines (Fig. 5, P, Q, S, and T). These results were consistent with the changes in nodule number in the *AsNIP43-*OE and RNAi transgenic plants.

We further performed RNA-seq analysis on *AsNIP43*-RNAi hairy roots inoculated with wild-type *M. huakuii* 7653R. Hairy roots transformed with empty vector were used as control (Supplemental Data Set 2). It showed that the expressions of defense response-related genes in the host at 1 dpi were remarkably increased. These DEGs in the *AsNIP43*-RNAi hairy roots included disease resistance protein, WRKY transcription factor, pathogenesis-related protein defense mechanisms, NBS-LRR resistance protein, disease resistance-like protein, and other defense response-related genes (Supplemental Table S4). We comparatively analyzed the KEGG pathway of the transcriptomes of *A. sinicus* inoculated with 7653R*ΔnopP* and *AsNIP43*-RNAi hairy roots. The results showed that both NopP and AsNIP43 are involved in the plant–pathogen interaction, isoflavone biosynthesis, flavonoid biosynthesis, nitrogen metabolism, plant hormone signal transduction, and other signaling pathways related to the symbiosis between rhizobia and legumes (Supplemental Fig. S9 and Table S5).

Taken together, the results indicated that *AsNIP43* is essential for rhizobial infection and may play an important role in the formation of infection threads and thus affects the nodule number. Besides, NopP and AsNIP43 may work and play roles in the same pathways.

### Symbiotic phenotype confirmation and characterization of homologous gene *MtRLK* in *M. truncatula*

**Figure 6.**
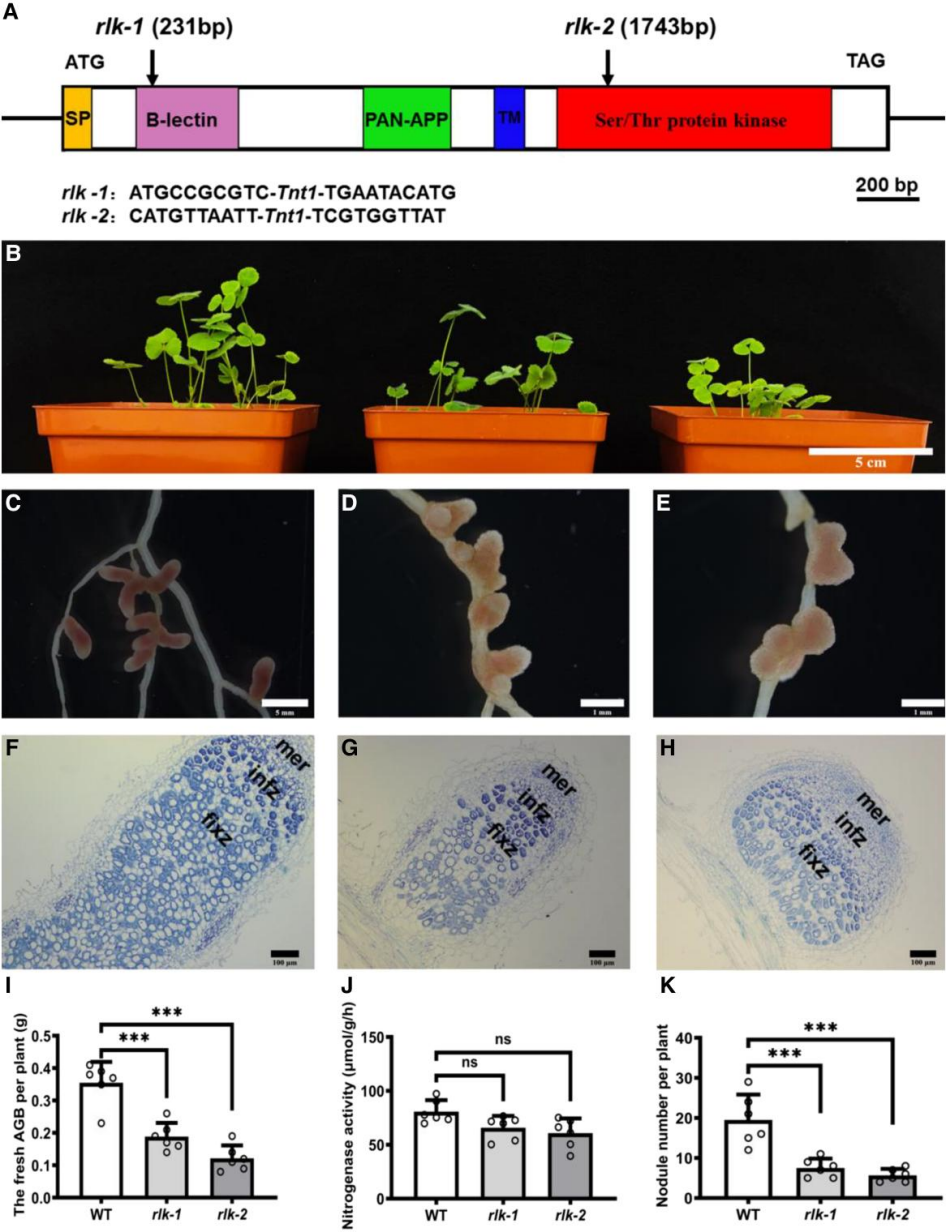
Symbiotic phenotypes of *AsNIP43* homolog gene (*MtRLK*) mutants. **A)** Insertion sites of *Tnt1* in the *MtRLK* gene. The mutant *rlk-1* (NF15380) had a *Tnt1* insertion in the B-lection domain, 231 bp after the ATG, whereas the mutant *rlk-2* (NF11649) contained a *Tnt1* insertion in the cytoplasmic kinase domain, 1,743 bp after the ATG. **B)** Aboveground biomass plant phenotypes, from left to right: WT *M. truncatula* and mutants *rlk-1*, *rlk-2*. **C to E**) Root nodule phenotypes. **F to H)** Paraffin sections of nodules from the WT **F**) and mutants **G and H**). mer, meristem zone; infz, infection zone; fixz, nitrogen fixation zone. **I to K**) Fresh aboveground biomass, nodule number per plant, and nitrogenase activity of WT and mutants (*n* = 6). Significance: ***P < 0.001; ns, not significant; Student's *t*-test. Data are mean ± Sd. The error bars represent the Sds of 3 independent experiments. Scale bars, 5 cm **B**), 5 mm **C**), 1 mm **D and E**), and 100 mm **F to H**).

**Figure 7.**
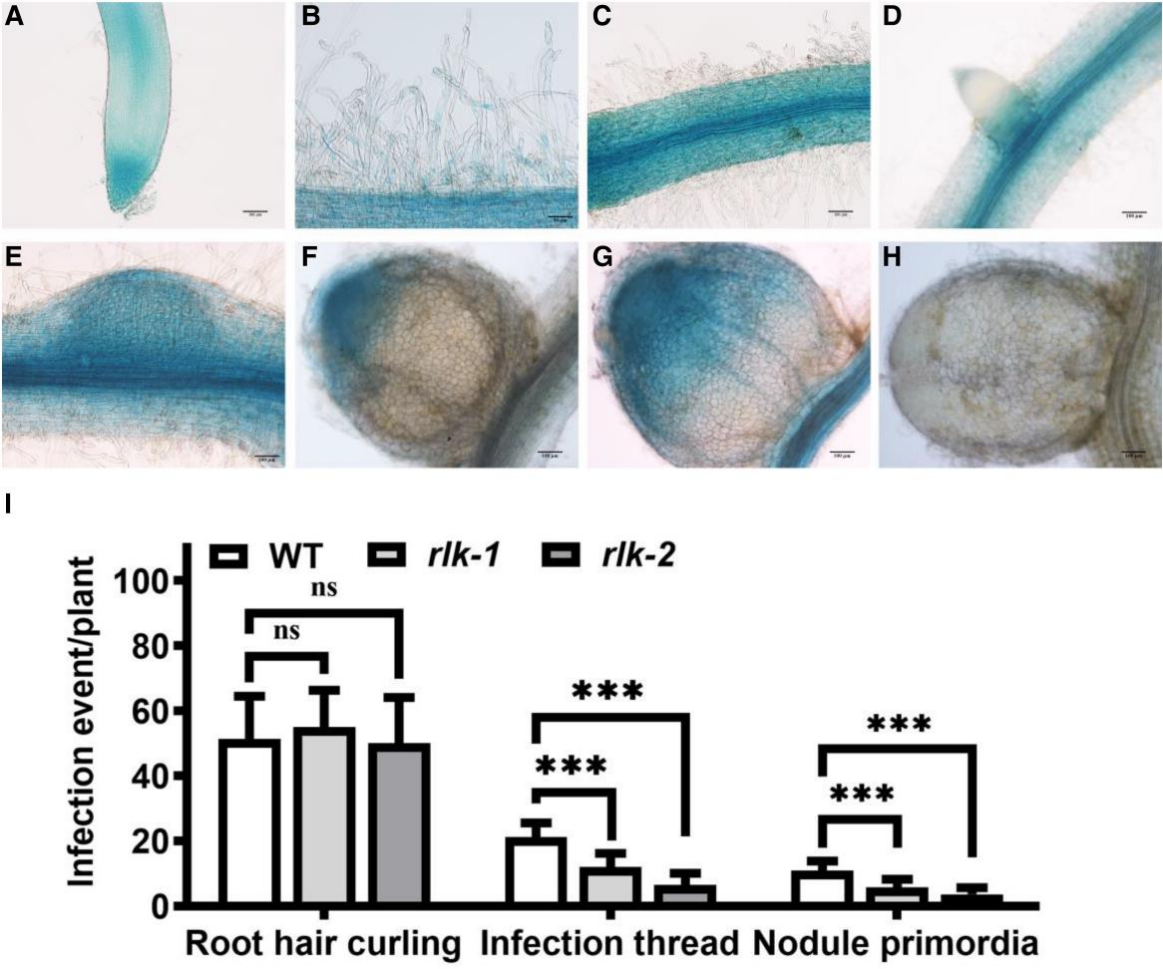
In situ expression pattern of *MtRLK* during infection and nodulation and infection events of *Mtrlk* mutants. Analysis of the expression pattern of *MtRLK* by GUS staining. **A** and **B)** Without the inoculation of *S. meliloti* 1021, *MtRLK* was highly expressed in root epidermal cells, root tips, and root hairs. **C to H)** After rhizobium inoculation, *MtRLK* was expressed in root epidermal cells and vascular tissues **C**), lateral root primordia **D**), nodule primordia **E**), infection zone of developing nodules **F and G**), but not in mature nodules **H**). **I)** Frequencies of early infection events per plant of *Mtrlk1* and *Mtrlk2* (*n* = 9). Scale bars, 50 *μ*m **B**); 100 *μ*m **A, C to H**). Significance: ***P < 0.001; ns, not significant; Student's *t*-test. Data are mean ± Sd. The error bars represent the Sds of 3 independent experiments.

To further confirm the symbiotic role of *AsNIP43*, a model legume *M. truncatula* was used for phenotype characterization. Based on the genome information, the *AsNIP43* putatively orthologous gene of *MtRLK* is *Medtr3g072800*. Similar to *AsNIP43*, *MtRLK* has no intron in the coding region and encodes a protein of 877 amino acids. MtRLK possesses the B-lectin, PAN-APP and conserved serine/threonine kinase domains as well (Fig. 6A). An analysis on the expression pattern of *MtRLK* (atlas database) showed that the *MtRLK* transcripts were detected in both noninoculated and inoculated roots, but were very low in mature nodules (Supplemental Fig. S10; Benedito et al. 2008). In addition, using the GUS reporter driven by the promoter of *MtRLK* in transgenic hairy roots, it was found that *MtRLK* was expressed in epidermal cells including root hairs of both noninoculated and inoculated roots, particularly in the infection zone of young nodules, but almost not detectable in mature nodules (Fig. 7, A to H). In brief, all these symbiotic expression patterns of *MtRLK* were generally the same as those of *AsNIP43*.

**Figure 8.**
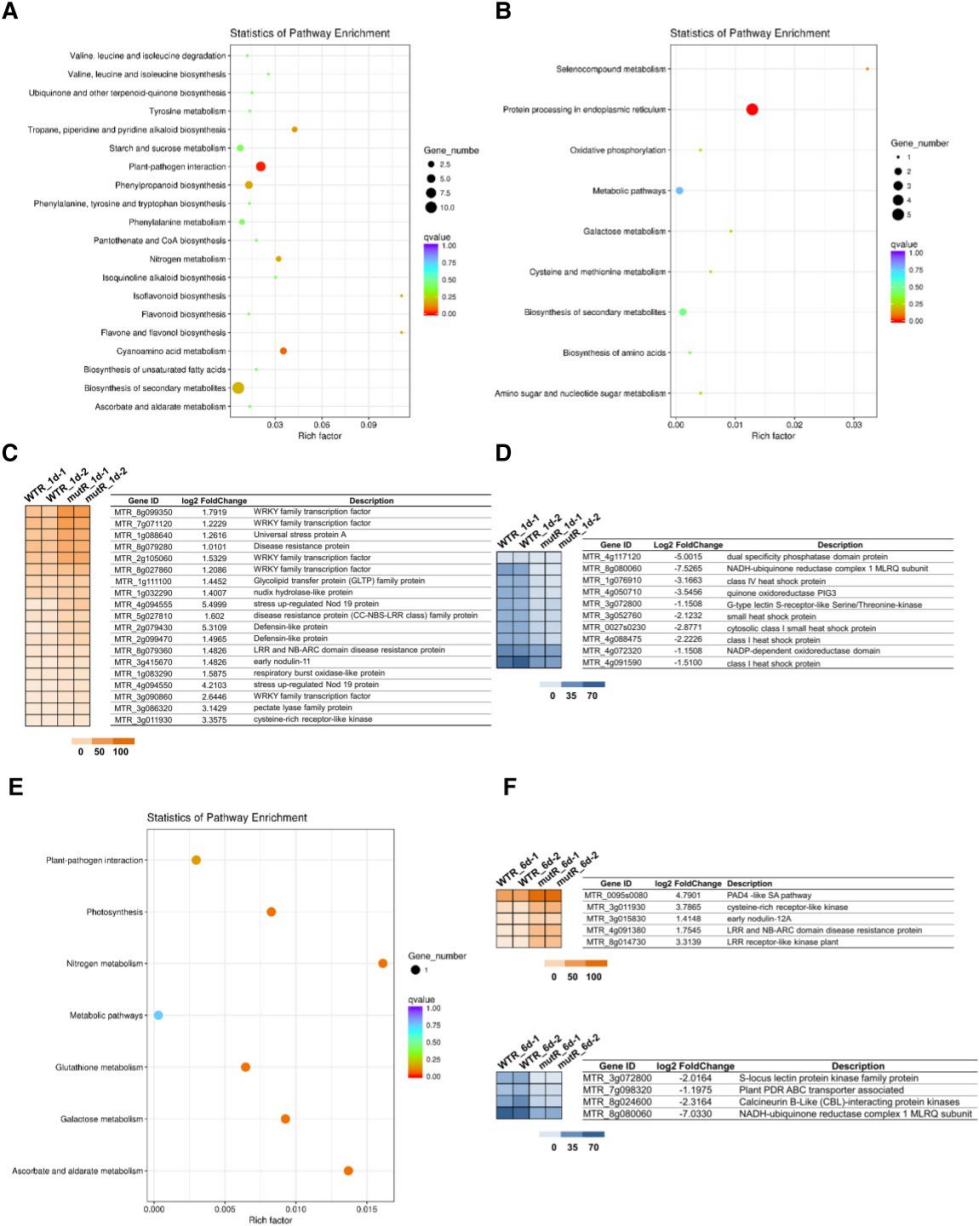
Effects of *Mtrlk* mutation on global gene expression in roots of *M. truncatula*. **A)** KEGG enrichment analysis of upregulated DEGs in the WTR_1d/mutR_1d group. **B)** KEGG enrichment analysis of downregulated DEGs in the WTR_1d/mutR_1d group. **C** and **D)** Hierarchical clustering analysis of the changes in upregulation and downregulation of symbiosis and defense genes in the WTR_1d/mutR_1d group. **E)** KEGG enrichment analysis of upregulated DEGs in the WTR_6d/mutR_6d group. **F** and **G)** Hierarchical clustering analysis of the changes in upregulation and downregulation of symbiosis and defense genes in the WTR_6d/mutR_6d group. The colored scales vary from light to dark, which indicate the levels of genes expression.

To analyze the symbiotic phenotypes, we employed a PCR-based reverse screening approach and obtained 2 independent *Tnt1* insertion *Mtrlk* mutants: NF15380 (*rlk-1*) containing a *Tnt1* insertion in the B-lectin domain, and NF11649 (*rlk-2*) with a *Tnt1* insertion in the serine/threonine kinase domain (Fig. 6A), both of which were null mutants. The symbiotic phenotypes of homozygous *Mtrlk* mutants and wild-type *M. truncatula* R108 plants at 28 dpi were examined (Fig. 6B). The results showed that both *Mtrlk* mutant lines had significantly fewer nodules and reduced shoot biomass per plant (Fig. 6, I to K). Notably, the nodules of the mutant lines were slightly pink and smaller in size (Fig. 6, C to E), with a smaller number of infected cells in the nodule. The histological analysis showed that the nodules were generally the same as the WT nodules in internal structure (Fig. 6, F to H). RNA-seq analysis was further performed to examine the global transcription profile and changes in *Mtrlk* mutant after inoculation with *Sinorhizobium meliloti* 2011 (Supplemental Data Set 3). We first analyzed the DEGs in the root (WTR_1d/muR_1d) at 1 dpi. Compared with the roots of WT plants, there were 185 DEGs (154 upregulated and 31 downregulated) in the mutant roots. Among them, those upregulated genes were substantially enriched in plant–pathogen interactions, biosynthesis of secondary metabolites, and other pathways, and the downregulated genes were substantially enriched in ER protein processing and other pathways (Fig. 8, A and B). The proteins encoded by these genes upregulated at 1 dpi included WRKY transcription factor, disease resistance (R) protein, defensin-like protein, stress protein, and respiratory burst oxidase-like protein (Fig. 8C). The upregulated expression of these genes in mutants indicated that the *MtRLK* gene may be involved in the defense response of alfalfa plants against the invasion of rhizobia during the early infection process. The proteins encoded by these downregulated genes included receptor-like kinases, heat shock proteins, and others (Fig. 8D). At 6 dpi, compared with the WT roots, there were 48 DEGs (40 upregulated and 8 downregulated) in the mutant roots. Among them, the proteins encoded by these upregulated genes were substantially enriched in plant–pathogen interaction, nitrogen metabolism, photosynthesis, and other pathways (Fig. 8E). The proteins encoded by these upregulated genes in the roots of the mutant at 6 dpi included R protein, SA signal pathway, and others, which are related to defense response (Fig. 8F). In short, transcriptome analysis indicated that knockdown of *Mtrlk* interrupts the coordinated gene expression of symbiosis and defense. Similarly, we also comparatively analyzed the KEGG pathway of the transcriptomes of *A. sinicus* inoculated with 7653R*ΔnopP* and *Mtrlk* roots. The results showed that both NopP and MtRLK are involved in the plant–pathogen interaction, isoflavone biosynthesis, flavonoid biosynthesis, nitrogen metabolism, and other signaling pathways related to the symbiosis between rhizobia and legumes (Supplemental Fig. S9 and Table S6). In addition, no difference was found between *Mtrlk* lines and WT R108 plants in root hair curling, but the number of infection threads and nodule primordia was significantly reduced in *rlk-1* and *rlk-2* (Fig. 7I). Taken together, all these results indicated that *MtRLK* functions similar to *AsNIP43* in legume-rhizobia symbiosis.

## Discussion

In this study, we identified and characterized the symbiotic function of a legume G-type lectin receptor-like kinase (LecRLK), AsNIP43, which interacts with the T3SS effector NopP from *M. huakuii* 7653R. Moreover, the symbiotic phenotypes were confirmed in the model legume *M. truncatula*. Our findings provide insights into the molecular mechanism for the involvement of LecRLKs and rhizobial effector NopP in the signaling pathway of the symbiotic interaction between rhizobia and legume.

LecRLKs are widely present in plants, but not in humans and yeast (Navarro-Gochicoa et al. 2003; Vaid et al. 2012). According to the lectin domains, LecRLKs are divided into 3 groups, including the G-type, L-type, and C-type (Shiu and Bleecker 2001). The G-type LecRLKs possess a α-D-mannose-specific lectin domain, and are also known as B-type LecRLKs because the N-terminal domain is similar to bulb-lectin proteins in humans and animals (Elola et al. 2015). G-type LecRLKs are also called S-domain LecRLKs due to the presence of the S-locus domain related to pollen self-incompatibility (Takasaki et al. 2000). Most G-type LecRLKs harbor the epidermal growth factor domain and the PAN domain (Vaid et al. 2012). The lectin domain is involved in carbohydrate-binding and the PAN domain is also involved in protein–carbohydrate and protein–protein interactions (Naithani et al. 2007; Bellande et al. 2017). In *Arabidopsis thaliana*, a lectin receptor kinase LecRK-I.9 was reported to target at the RXLR effector of *Phytophthora infestans* and disturbs infection (Bouwmeester et al. 2011). And an L-type lectin receptor-like kinase LecRK-IX.2 can phosphorylate the AvrPtoB virulence effector of *Pseudomonas syringae* and suppress PTI in *Arabidopsis* (Xu et al. 2020). LORE, a G-type lectin receptor-like kinase in *Arabidopsis* can recognize bacterial medium-chain 3-hydroxy fatty acids (mc-3-OH-FAs), and subsequently trigger immunity response (Kutschera et al. 2019; Luo et al. 2020). Another G-type lectin receptor-like kinase from *N. benthamiana*, ERK1, was reported to regulate the perception of an apoplastic effector EXLX1 from *Phytophthora capsici* and positively mediate the plant resistance (Pi et al. 2022). Rhizobial effector NopP is secreted into host legume cells through T3SS, which is generally considered as a determinant of symbiotic incompatibility to regulate the range of host plants (Sugawara et al. 2018). In this work, a legume G-type LecRLK AsNIP43, interacting with the rhizobial T3SS effector NopP, was reported and characterized. AsNIP43 is a G-type lectin receptor-like kinase, with the typical N terminus of SP, B-lectin, S-locus, and PAN/APP, an intermediate transmembrane domain and a C-terminal Ser/Thr kinase domain (Elola et al. 2015). The symbiotic phenotype analysis revealed that AsNIP43 is essential for rhizobial infection and positively regulates root nodule number.

Notably, *AsNIP43* plays a role in the formation of infection threads. Expression pattern analysis revealed that *AsNIP43* is expressed in epidermal cells and root hairs, with particularly high expression in the nodule primordia and infection zone of young nodules, but not in mature nodules. Inoculation of GFP-labeled 7653R revealed that the number of infection threads and nodule primordia was reduced in *AsNIP43* RNAi roots, while the *AsNIP43* overexpression roots had more infection threads and nodule primordia. Therefore, it can be speculated that *AsNIP43* plays a role in the formation of infection threads and positively regulate symbiosis. MtLecRK1;1, a lectin-like receptor kinase in *M. truncatula*, has higher expression in the roots and its expression level is increased by nitrogen starvation, but suppressed by rhizobial inoculation or NF treatment. Molecular structure modeling showed that MtLecRK1;1 can interact with NFs and overexpression of *MtLecRK1;1* in roots would lead to an increase in root nodule number, indicating that MtLecRK1;1 is involved in the symbiotic nodulation process (Navarro-Gochicoa et al. 2003). The expression pattern and symbiotic phenotype of *MtRLK* in *M. truncatula* were similar to those of *AsNIP43*. In addition, transcriptome analysis indicated that *Mtrlk* knockdown resulted in global increases in the expression of defense genes that affected symbiosis. Therefore, AsNIP43 positively promotes the symbiosis.

Characterization of the symbiotic phenotypes of mesorhizobia NopP in this work showed that *M. huakuii* 7653R NopP functions during the infection of rhizobia, negatively regulates the nodule number. In this work, *nopP* expression was detected in root hairs at 3 d after rhizobial inoculation, particularly in the infection zone of young nodules, but not in mature nodules, and at the same time, the number of infection threads and nodule primordia increased under inoculation with 7653RΔ*nopP*. The similar expression patterns of *AsNIP43* and *nopP* implied that they might function together during the infection process of symbiosis. Previous studies have reported that the *nopP* mutation has a positive, negative, or no impact on symbiosis depending on the legume host plants (Ausmees et al. 2004). Mutation of *nopP* in NGR234 resulted in a significant reduction in nodule numbers with *F. congesta* and *T. vogelii*, while showed little effect on *Pachyrhizus tuberosus*. In soybean, NopP of Bradyrhizobia possibly interacts with the Rj2 protein directly or indirectly, acting as the determinant of symbiotic incompatibility (Sugawara et al. 2018). Here, we found that the T3SS effector NopP of *M. huakuii* 7653R may negatively regulate the symbiotic relationship with the host plant *A. sinicus* by initiating defense responses. Meanwhile, RNA-seq data of *AsNIP43*-RNAi hairy roots suggest that AsNIP43 may promote symbiosis by affecting the genes expression related with early defense responses. We compared the enriched KEGG pathway based on the DEGs of the 2 group RNA-seq, it showed that NopP and AsNIP43 may work in the same pathway that related to symbiosis between rhizobia and legumes. In a recent publication, we reported that an R protein of soybean interacts with the T3SS effector NopP of USDA110 to restrict nodulation under incompatible symbiosis (Zhang et al. 2021). We speculate that there may also be an unknown R protein in *A. sinicus*. The AsNIP43 interacting with NopP may divert the signaling pathway, and therefore weaken or block the usual ETI reaction, which usually mediates by in an unknown R protein. As a result, NopP–AsNIP43 interaction will enhance symbiotic nodulation. However, when the *nopP* gene of *M. huakuii* 7653R is deleted, the ETI response is attenuated and therefore enhances symbiosis (Supplemental Fig. S11).

Taken together, we identified a target of T3SS effector NopP of *M. huakuii* 7653R in legume *A. sinicus*. The findings deepen our understanding on the functions of rhizobial effector NopP and its legume-interacting protein in the establishment of nodule symbiosis.

## Materials and methods

### Plant materials and growth conditions

Chinese milk vetch (*A. sinicus*) was used for symbiotic phenotype analysis and hairy root transformation. The seeds were surface sterilized in 75% (v/v) ethanol for 15 min, and then treated with 5% sodium hypochlorite for 15 min. To synchronize the germination, the sterilized seeds were immersed in distilled water for 24 h, and then placed on 0.5% (w/v) sucrose and 0.55% (w/v) agar plates. Seedlings were grown under a 16/8-h light/dark cycle at 25°C/18°C, and then were transferred into sterile sand and watered with Fahraeus nitrogen-free plant nutrient solution (Lei et al. 2014). *N. benthamiana* was grown in a growth chamber at 22°C/16°C with a day/night photoperiod of 16/8 h. After 3 to 4 wk, the leaves were used for transient protein expression. The infiltrated plants were maintained under the same growth conditions. *M. truncatula* cv. Jemolong A17 was used for hairy root transformation, and the *M. truncatula* ssp. *tricycla* R108 and 2 *Tnt1* insertion mutants (NF15380 and NF11649) were used for symbiotic phenotype analysis. The *Tnt1* lines were available at Noble Research Institute and screened by a nested PCR approach (Tadege et al. 2008). Seeds were soaked in H_2_SO_4_ for 2 to 3 min, and then sterilized with 2% (w/v) sodium hypochlorite for 3 to 5 min. The surface sterilized seeds were immersed in distilled water for 2 d in the dark at 4°C for synchronization, and then transferred onto Fahraeus nitrogen-free medium with 1.2% (w/v) agar for germination at 25°C in the dark. The seedlings were placed into sterile sand and watered with Fahraeus nutrient solution. Plants were grown in a growth chamber at 24°C/18°C with a 16/8-h day/night cycle.

### Bacterial strains and growth conditions

The bacterial strains used in this study are listed in Supplemental Table S1. Wild-type *S. meliloti* 2011 and *M. huakuii* 7653R, and strains derived from 7653R were grown in Tryptone Yeast medium at 28°C. *Escherichia coli* DH5a and *E. coli* Rosetta (DE3) were cultured in Luria-Bertani (LB) medium at 37°C. *Agrobacterium rhizogenes* K599 was used for hairy root transformation of *A. sinicus* while *A. rhizogenes* MSU440 was used for hairy root transformation of *M. truncatula*, both of which were cultured in LB medium at 28°C. *Agrobacterium tumefaciens* strain GV3101 was used for transient expression of proteins in *N. benthamiana* leaves, and grown in LB medium at 28°C. The following concentrations of antibiotics were used for the above strains: streptomycin, 100 *μ*g mL^−1^; kanamycin, 50 *μ*g mL^−1^; gentamycin, 25 *μ*g mL^−1^; ampicillin, 100 *μ*g mL^−1^; spectinomycin, 100 *μ*g mL^−1^; rifampicin, 100 *μ*g mL^−1^; tetracycline, 12.5 *μ*g mL^−1^; chloromycetin, 34 *μ*g mL^−1^.

### Construction of *nopP* mutant and complementary strains

Primers used for DNA amplification in this study are listed in Supplemental Table S2. The *cre-lox* system was used to construct the in-frame 7653R *nopP* gene deletion mutant. The fragments containing the flanking sequences of the upstream N-terminal coding region and the downstream C-terminal coding region of *nopP* were cloned into the *Nco*I/*Nde*I and *Apa*I/*Age*I sites of pCM184. The recombinant plasmid was conjugated into *M. huakuii* 7653R by bi-parental mating. To eliminate the kanamycin resistance gene flanked by *loxP* sites, the plasmid pCM157 expressing Cre recombinase was introduced into 7653RΔ*nopP*::Kan by conjugation. The transconjugants sensitive to kanamycin were screened and then the strains sensitive to tetracycline were selected. The candidates were confirmed by PCR and sequencing. A strain with a verified deletion in the *nopP* gene was named as 7653RΔ*nopP*.

To construct the recombinant plasmid expressing NopP, the fragment containing *nopP* coding sequence and its upstream promoter region was cloned into the *Bam*HI/*Hin*d III sites of pBBR1MCS-5 in the opposite orientation to the *lac* promoter, thereby preventing nonspecific transcription. The resultant pBBR1MCS-5-*nopP* was transformed into 7653RΔ*nopP* by bi-parental mating. The complementary strain obtained was named as 7653RΔ*nopP*-C. For the overexpression vector, the ORF of *nopP* was inserted into the *Hin*d III and *Bam* HI sites of the pBBR1MCS-5, in the same orientation as the *lac* promoter, the *lac* promoter was used to drive the expression of the *nopP*. The recombinant plasmid was introduced into wild-type *M. huakuii* 7653R by conjugation, and the resulting strain was named as 7653R*nopP*-O.

### Yeast 2-hybrid screening and interaction study

The full-length 7653R *nopP* was amplified and cloned into the EcoRI/BamHI sites of the vector of pGBKT7. The bait was used to screen the *A. sinicus* cDNA library for the interacting clones according to the manufacturer's instructions (CLONTECH). For the construction of the truncated segments of AsNIP43 fused to GAL4 AD proteins, the fragments encoding B-lectin (67 to 477 bp), S-locus (67 to 1,008 bp containing B-lectin), PAN (67 to 1,317 bp encompassing B-lectin and S-locus), and S-TKs (1,504 to 2,196 bp) were cloned into the *Eco*RI/*Bam*HI sites of pGADT7. To verify the protein–protein interactions in *Saccharomyces cerevisiae* cells, BD fusion plasmid and AD fusion plasmid were transformed into the strains Y187 and AH109, respectively. After mating of the 2 strains, the growing colonies on the SD/-Leu-Trp plates were transferred to SD/-Leu-Trp/X-gal and SD/-Leu-Trp-Ade-His/X-gal plates for further examination of interaction. β-Galactosidase activity were assayed according to the previous method (Zhu et al. 2008).

### Protein expression and purification

For in vitro interaction assays, the 7653R *nopP* ORF was cloned into the *Nde*I/*Bam*HI sites of the expression vector pET28a to express the 6×His-NopP fusion protein. The PAN region of AsNIP43 was introduced into the *Sma*I/*Xho*I sites of the expression vector pGEX6P-1 for expressing the GST-PAN fusion protein. For the expression of fusion proteins, the *E. coli* Rosetta (DE3) harboring the corresponding plasmids was cultured overnight at 20°C with 0.5 mM IPTG. His-tagged proteins were purified using native IMAC cartridge (BIO-RAD). GST fusion proteins were purified using glutathione resin (Genscript). All the purification procedures were performed according to the manufacturer's instructions.

### In vitro protein–protein interaction

To confirm protein interaction in vitro, equal amounts of the purified GST-PAN and GST protein were recombined on the glutathione resin, which were then incubated with an equal amount (20 *μ*g) of purified His-NopP in the interaction buffer (140 mM NaCl, 2.7 mM KCl, 10 mM Na_2_HPO_4_, 1.8 mM KH_2_PO_4_, 0.1% Triton X-100, pH 7.4). The mixture were incubated in the interaction buffer on ice for 2 h with gentle shaking, and centrifuged at 4°C, 2,000 rpm for 1 min. After incubation and centrifugation, the resin was washed with wash buffer (140 mM NaCl, 2.7 mM KCl, 10 mM Na_2_HPO_4_, 1.8 mM KH_2_PO_4_, 0.1% Triton, pH 7.4). The samples were added with 1×SDS loading buffer and separated by an SDS–PAGE gel, followed by immunoblotting detection with HRP-conjugated anti-His antibody.

### BiFC assays in *N. benthamiana* leaves

For the BiFC experiment, the 7653R *nopP* without the stop codon was cloned into the *Sal*I/*Bam*HI sites of pXY106-nYFP. The full-length AsNIP43 and PAN region fragments were cloned into the *Sal*I/*Bam*HI sites of pXY104-cYFP. For infiltration of the leaves, the strain GV3101 carrying the corresponding plasmids was cultured overnight until OD_600_ to 1.0. The cultures were collected and re-suspended in infiltration buffer (10 mM MES-OH, pH 6.0, 10 mM MgCl_2_, 200 *μ*M acetosyringone). For the co-expression of proteins, equal volumes of 2 GV3101 strains were mixed and infiltrated into the leaves of *N. benthamiana*. At 48 to 60 h after infiltration, the leaves were observed by a laser scanning confocal microscope.

### CoIP assays

For CoIP assays, the *AsNIP43* without the stop codon was amplified and cloned into the *Nco*I/*Spe*I sites of pCAMBIA1302-eGFP. The 7653R *nopP* without the stop codon was cloned into the *Xba*I/*Kpn*I sites of pUB-3×HA. HA-NopP and GFP-AsNIP43 or GFP were co-expressed in *N. benthamiana* leaves. Seventy-two hours after infiltration, the leaves were ground into powder in liquid nitrogen, and 1 g leaves were added with 1 mL extraction buffer to extract proteins. The samples were solubilized with extraction buffer (50 mM Tris–HCl pH 7.4, 150 mM NaCl, 1 mM EDTA pH 8.0, 1% Triton X-100 (v/v), 5% glycerol (v/v), and protease inhibitor cocktail). The extracts were centrifuged and the supernatant was collected and incubated with anti-GFP agarose beads at 4°C for 3 h. The beads were washed and the samples were separated by SDS–PAGE, followed by immunoblotting analysis with anti-GFP and anti-HA antibodies (ABclonal Technology).

### Subcellular localization of proteins in *N. benthamiana* leaves

For subcellular localization analysis, the 7653R *nopP* gene and the *AsNIP43* gene without stop codons were amplified and cloned into the *Nco*I/*Spe*I restriction sites of pCAMBIA1302-eGFP vector. The NopP-eGFP and AsNIP43-eGFP constructs were co-expressed with the plasma membrane marker CERK1-DsRed or the ER marker HDEL-mCherry, respectively, in the epidermal cells of *N. benthamiana*. For co-localization, the coding region of *nopP* was amplified and inserted into the *Nco*I/*Spe*I restriction sites of pCAMBIA1302-DsRed. The NopP-DsRed and AsNIP43-eGFP constructs were co-expressed in *N. benthamiana* as described above. The samples were observed at 48 to 60 h after infiltration by a confocal microscope.

### RNAi and overexpression of *AsNIP43* in *A. sinicus*

For RNAi experiment, to generate *AsNIP43*-RNAi lines, a 215-bp fragment of the 3′ UTR of *AsNIP43* was amplified (primers, see Supplemental Table S1) and cloned into the pDONR221 vector, and then recombined into the pK7GWIWG2D(II)-RootRed vector (Karimi et al. 2002). The empty vector Cheap-RNAi was used as the control. For *AsNIP43* overexpression construct, the full-length of *AsNIP43* was amplified and cloned into the *Xba*I/*Sac*I sites of pBI121-GFP and under the control of the CaMV35S promoter. The vectors were transformed into *A. rhizogenes* K599.

*A. sinicus* transformation mediated by *A. rhizogenes* K599 was performed as described previously (Lei et al. 2014). The positive plants were confirmed by fluorescence from DsRed and GFP reporter in transgenic hairy roots. Only the strong fluorescence roots were chosen for further studies. Then, 1 to 3 positive roots were left for each plant, which was then transplanted into sterilized sand. After 7 to 9 d, the transgenic plants were inoculated with *M. huakuii* 7653R.

### Promoter GUS analysis

A 600-bp fragment upstream the *nopP* was selected as the putative promoter region, and then amplified from *M. huakuii* 7653R genomic DNA by PCR using the primers *nopP*-P-F and *nopP*-*P*-R. The amplicons were cloned into the *Sma*I and *Pst*I site of pGR960 to create the pGR960-*nopP*-GUS construct. The recombinant plasmid was transformed into wild-type *M. huakuii* 7653R. To obtain the *AsNIP43* promoter fragment upstream of the translation initiator from *A. sinicus* genomic DNA, primers (Supplemental Table S2) were used for PCR amplification. The amplified fragments were purified and cloned into the *Bam*HI and *Hin*d III site of DX2181GFPa, resulting in a recombinant construct DX2181GFP-p*AsNIP43*-GUS. The construct containing the promoter fusion was transformed into *A. rhizogenes* K599, and then transformed into *A. sinicus* by *A. rhizogenes*-mediated transformation. A 1,563 bp putative promoter region immediately upstream of the start codon of *MtRLK* was amplified from *M. truncatula* genomic DNA, and cloned into the *Bam*HI and *Hin*d III site of DX2181GFPa. The recombinant construct was transformed into *A. rhizogenes* MSU440, and the *A. rhizogenes*-mediated hairy root transformation of *M. truncatula* was performed by a standard procedure (Medicago Handbook).

The histochemical staining of GUS activity was performed according to previous descriptions (Wilson et al. 1995). Roots and nodules were stained using GUS-staining buffer (0.1 M sodium phosphate buffer pH 7.0, 2 mM K_4_Fe (CN)_6_, 2 mM K_4_Fe (CN)_6_, 0.1% (v/v) Triton X-100, 10 mM EDTA, 0.5 mg mL^−1^ X-Gluc dissolved in DMF). The root and nodule sections were observed by a light microscope (BX51, Olympus, Japan) to visualize the spatial GUS activity.

### Phylogenetic analysis

The phylogenetic analysis of NopP and AsNIP43 was performed by MEGA7 software (version 7.0) (Kumar et al. 2016). Homologous sequences of NopP and AsNIP43 were searched through the BLAST programs (https://blast.ncbi.nlm.nih.gov/Blast.cgi). The multiple protein sequences were aligned using ClustalO, and the aligned sequences were loaded to MEGA7.0 for the generation of the phylogenetic tree. The evolutionary history was inferred using the Neighbor-Joining method.

### Nitrogenase activity measurement

Nitrogenase activity was measured by acetylene reduction activity method as described previously (Zhou et al. 2019).

### Confocal microscopy

Confocal microscopy analysis was performed by an Olympus FV1000 laser scanning confocal microscope. In the BiFC experiment, the excitation wavelength used to detect YFP was 514 nm, and the emitted fluorescence was collected from 522 to 558 nm. In subcellular localization and colocalization experiments, the excitation wavelength used to detect GFP was 488 nm, while the emission was detected at 500 to 550 nm. The DsRED was excited at 543 nm, while the emitted fluorescence was detected at 565 to 615 nm for DsRED.

### Accession numbers

Sequence information in this article can be found in the GenBank libraries and plant genome according to the following accession numbers: NopP (QGU20929.1), AsNIP43 (MT435087). The NopP homologs sequences for phylogenetic analysis were: *Sinorhizobium fredii* HH103 (AAY33495.1), *S. fredii* CCBAU 45436 (WP_037457110.1), *S. fredii* USDA 257 (AFL55005.1), *S. fredii* CCBAU 05631 (ASY60917.1), *S. fredii* NGR234 (P55724.1), *Sinorhizobium* sp. BJ1 (WP_097523465.1), *Sinorhizobium* sp. PC2 (WP_046119641.1), *Mesorhizobium delmotii* (WP_165848632.1), *Mesorhizobium prunaredense* (WP_077376236.1), *Mesorhizobium* sp. M1A.F.Ca.IN.022.07.1.1 (WP_127323344.1), *Mesorhizobium* sp. M6A.T.Ce.TU.002.03.1.1 (WP_127204235.1), *Mesorhizobium tamadayense* (WP_125005402.1), *Mesorhizobium waimense* (WP_120019108.1), *Mesorhizobium sophorae* (WP_095082018.1), *M. amorphae* (WP_100084785.1), *Bradyrhizobium* sp. ORS 3257 (SPP98457.1), *Bradyrhizobium shewense* (WP_091956564.1), *B. elkanii* (WP_069277638.1), *B. diazoefficiens* USDA122 (AND87361.1), *Bradyrhizobium* sp. CCBAU 15635 (WP_038973567.1), *B. diazoefficiens* USDA 110 (AND87361.1), *Bradyrhizobium liaoningense* (WP_035678775.1), *Bradyrhizobium japonicum* USDA 6 (AGH09949.1). The AsNIP43 homologs for phylogeny tree: *Glycine soja* (XP_028235849.1:12-875), *G. max* (NP_001238617.1:1-771), *Vigna angularis* (XP_017421053.1:11-860), *P. vulgaris* (XP_007137381.1:1-859), *Cajanus cajan* (XP_020225818.2:1-857), *Mucuna pruriens* (RDX84078.1:1-857), *M. truncatula* (XP_003601079.1:3-879), *Lupinus albus* (KAE9587183.1:1-861), *Lupinus angustifolius* (XP_019433154.1:1-859), *Prosopis alba* (XP_028757505.1:16-853), *Arachis duranensis* (XP_015934757.1:20-898), *Nicotiana tabacum* (XP_016462282.1:1-870), *Morus notabilis* (XP_010091359.1:1-863), *Trema orientale* (PON80868.1:48-865), *Gossypium arboreum* (XP_017645457.1:15-869), *Manihot esculenta* (XP_021603744.1:1-860), *Zea mays* (XP_008646310.1), *Ricinus communis* (XP_002513778.2:6-864), *A. thaliana* (NP_198387.2), *Oryza sativa* (XP_015612247.1:38-887), and *N. benthamiana* (BAG68210.1:323-664).

## Supplementary Material

kiad318_Supplementary_Data

## Data Availability

The authors confirm that all experimental data are available.

## Supplemental data

The following materials are available in the online version of this article.

**Supplemental Methods S1.** Supplemental materials and methods describing secretion assay, construction, and screening of *A. sinicus* yeast 2-hybrid cDNA library, observation and quantification of early infection events, RT-qPCR, and RNA-seq.

**Supplemental Figure S1.** T3SS and effector NopP of *M. huakuii* 7653R.

**Supplemental Figure S2.** Phylogenetic analysis of NopP homologs.

**Supplemental Figure S3.** Effects of 7653R*nopP* on rhizobial growth under free-living condition.

**Supplemental Figure S4.** Expression levels of the *nopP* in different mutants under free-living condition.

**Supplemental Figure S5.** KEGG pathway enrichment analysis of DEGs in host inoculated with 7653RΔ*nopP*.

**Supplemental Figure S6.** Sequence alignment of AsNIP43 and homologs from other legume plants.

**Supplemental Figure S7.** Phylogenetic analysis of AsNIP43 homologs.

**Supplemental Figure S8.** Expression levels of the *AsNIP43* and *nopP* genes at different time points after inoculation.

**Supplemental Figure S9.** Venn diagrams showing the comparative analysis KEGG pathways in RNA-seq of *A. sinicus* roots inoculated with 7653R*ΔnopP* and AsNIP43 RNAi hairy roots, and *A. sinicus* roots inoculated with 7653R*ΔnopP* and *Mtrlk* mutant roots.

**Supplemental Figure S10.** Expression profiles of *MtRLK* gene in the database of Bio-Analytic Resource.

**Supplemental Figure S11.** Model for the regulation of a LecRLK AsNIP43 on the symbiosis in *A. sinicus*.

**Supplemental Table S1.** The strains and plasmids used in this study.

**Supplemental Table S2.** Primers used in this study.

**Supplemental Table S3.** Candidate interacting proteins from NopP bait screening of *A. sinicus* cDNA library.

**Supplemental Table S4.** DEGs related to defense response in CK vs AsNIP43-RNAi at 1 dpi.

**Supplemental Table S5.** The common KEGG pathways in RNA-seq of *A. cinisus* roots inoculated with 7653R*ΔnopP* and AsNIP43 RNAi hairy roots.

**Supplemental Table S6.** The common KEGG pathways in RNA-seq of *A. sinicus* roots inoculated with 7653R*ΔnopP* and *Mtrlk* mutant roots.

**Supplemental Data Set 1.** The FPKM of all genes in *A. sinicus* roots at 1 and 6 dpi with wild-type *M. huakuii* 7653R and *M. huakuii* 7653R-*ΔnopP*.

**Supplemental Data Set 2.** The FPKM of all genes in *A. sinicus* of AsNIP43-RNAi hairy roots at 1 and 7 dpi with wild-type *M. huakuii* 7653R.

**Supplemental Data Set 3.** The FPKM of all genes in *M. truncatula* wild-type (R108) and *Mtrlk* mutant roots at 1 and 6 dpi with wild-type *S. meliloti* 2011.

## References

[kiad318-B1] Ausmees N , Kobayashi H, Deakin WJ, Marie C, Krishnan HB, Broughton WJ, Perret X. Characterization of NopP, a type III secreted effector of *Rhizobium* sp. strain NGR234. J Bacteriol. 2004:186(14):4774–4780. 10.1128/JB.186.14.4774-4780.2004 15231809 PMC438593

[kiad318-B2] Batistic O , Waadt R, Steinhorst L, Held K, Kudla J. CBL-mediated targeting of CIPKs facilitates the decoding of calcium signals emanating from distinct cellular stores. Plant J. 2010:61(2):211–222. 10.1111/j.1365-313X.2009.04045.x 19832944

[kiad318-B3] Bellande K , Bono JJ, Savelli B, Jamet E, Canut H. Plant lectins and lectin receptor-like kinases: how do they sense the outside? Int J Mol Sci. 2017:18(6):1164–1189. 10.3390/ijms18061164 28561754 PMC5485988

[kiad318-B4] Benedito VA , Torres-Jerez I, Murray JD, Andriankaja A, Allen S, Kakar K, Wandrey M, Verdier J, Zuber H, Ott T, et al A gene expression atlas of the model legume *Medicago truncatula*. Plant J. 2008:55(3):504–513. 10.1111/j.1365-313X.2008.03519.x 18410479

[kiad318-B5] Bouwmeester K , de Sain M, Weide R, Gouget A, Klamer S, Canut H, Govers F. The lectin receptor kinase LecRK-I.9 is a novel Phytophthora resistance component and a potential host target for a RXLR effector. PLoS Pathog. 2011:7(3):e1001327–e1001339. 10.1371/journal.ppat.1001327 21483488 PMC3068997

[kiad318-B6] Cao Y , Halane MK, Gassmann W, Stacey G. The role of plant innate immunity in the legume-rhizobium symbiosis. Annu Rev Plant Biol. 2017:68(1):535–561. 10.1146/annurev-arplant-042916-041030 28142283

[kiad318-B7] Cho HJ , Yong X, Murooka Y. Formation of adventitious shoots and plant regeneration by culture of cotyledon segment in *Astragalus sinicus* (Chinese milk vetch). Plant Tissue Cult Lett. 1995:12(1):87–90. 10.5511/plantbiotechnology1984.12.87

[kiad318-B8] Eichinger V , Nussbaumer T, Platzer A, Jehl MA, Arnold R, Rattei T. EffectiveDB—updates and novel features for a better annotation of bacterial secreted proteins and type III, IV, VI secretion systems. Nucleic Acids Res. 2016:44(D1):D669–D674. 10.1093/nar/gkv1269 26590402 PMC4702896

[kiad318-B9] Elola MT , Blidner AG, Ferragut F, Bracalente C, Rabinovich GA. Assembly, organization and regulation of cell-surface receptors by lectin-glycan complexes. Biochem J. 2015:469(1):1–16. 10.1042/BJ20150461 26173257

[kiad318-B10] Ferguson BJ , Mens C, Hastwell AH, Zhang MB, Su H, Jones CH, Chu XT, Gresshoff PM. Legume nodulation: the host controls the party. Plant Cell Environ. 2019:42(1):41–51. 10.1111/pce.13348 29808564

[kiad318-B11] Ge YY , Xiang QW, Wagner C, Zhang D, Xie ZP, Staehelin C. The type 3 effector NopL of *Sinorhizobium* sp. strain NGR234 is a mitogen-activated protein kinase substrate. J Exp Bot. 2016:67(8):2483–2494. 10.1093/jxb/erw065 26931172

[kiad318-B12] Karimi M , Inzé D, Depicker A. GATEWAY vectors for *Agrobacterium*-mediated plant transformation. Trends Plant Sci. 2002:7(5):193–195. 10.1016/S1360-1385(02)02251-3 11992820

[kiad318-B13] Kawaharada Y , Kelly S, Nielsen MW, Hjuler CT, Gysel K, Muszyński A, Carlson RW, Thygesen MB, Sandal N, Asmussen MH, et al Receptor-mediated exopolysaccharide perception controls bacterial infection. Nature. 2015:523(7560):308–312. 10.1038/nature14611 26153863

[kiad318-B14] Kawaharada Y , Nielsen MW, Kelly S, James EK, Andersen KR, Rasmussen SR, Füchtbauer W, Madsen LH, Heckmann AB, Radutoiu S, et al Differential regulation of the Epr3 receptor coordinates membrane-restricted rhizobial colonization of root nodule primordia. Nat Commun. 2017:8(1):14534–14545. 10.1038/ncomms14534 28230048 PMC5331223

[kiad318-B15] Kumar S , Stecher G, Tamura K. MEGA7: molecular evolutionary genetics analysis version 7.0 for bigger datasets. Mol Biol Evol. 2016:33(7):1870–1874. 10.1093/molbev/msw054 27004904 PMC8210823

[kiad318-B16] Kutschera A , Dawid C, Gisch N, Schmid C, Raasch L, Gerster T, Schäffer M, Smakowska-Luzan E, Belkhadir Y, Vlot AC, et al Bacterial medium-chain 3-hydroxy fatty acid metabolites trigger immunity in *Arabidopsis* plants. Science 2019:364(6436):178–181. 10.1126/science.aau1279 30975887

[kiad318-B17] Lei L , Chen L, Shi X, Li Y, Wang J, Chen D, Xie F, Li Y. A nodule-specific lipid transfer protein AsE246 participates in transport of plant-synthesized lipids to symbiosome membrane and is essential for nodule organogenesis in Chinese milk vetch. Plant Physiol. 2014:164(2):1045–1058. 10.1104/pp.113.232637 24367021 PMC3912078

[kiad318-B18] Liu D , Luo Y, Zheng X, Wang X, Wei G. TRAPPC13 is a novel target of *Mesorhizobium amorphae* type III secretion system effector NopP. Mol Plant Microbe Interact. 2021:34(5):511–523. 10.1094/MPMI-12-20-0354-FI 33630651

[kiad318-B19] Luo X , Wu W, Liang Y, Xu N, Liu J. Tyrosine phosphorylation of the lectin receptor like kinase LORE regulates plant immunity. EMBO J. 2020:39(4):102856–102872. 10.15252/embj.2019102856 PMC7024837 31922267

[kiad318-B20] Madsen LH , Tirichine L, Jurkiewicz A, Sullivan JT, Heckmann AB, Bek AS, Ronson CW, James EK, Stougaard J. The molecular network governing nodule organogenesis and infection in the model legume *Lotus japonicus*. Nat Commun. 2010:1(1):1–12. 10.1038/ncomms1009 20975672 PMC2892300

[kiad318-B21] Miwa H , Okazaki S. How effectors promote beneficial interactions. Curr Opin Plant Biol. 2017:38:148–154. 10.1016/j.pbi.2017.05.011 28622658

[kiad318-B22] Miya A , Albert P, Shinya T, Desaki Y, Ichimura K, Shirasu K, Narusaka Y, Kawakami N, Kaku H, Shibuya N. CERK1, a LysM receptor kinase, is essential for chitin elicitor signaling in *Arabidopsis*. Proc Natl Acad Sci U S A. 2007:104(49):19613–19618. 10.1073/pnas.0705147104 18042724 PMC2148337

[kiad318-B23] Naithani S , Chookajorn T, Ripoll DR, Nasrallah JB. Structural modules for receptor dimerization in the *S*-locus receptor kinase extracellular domain. Proc Natl Acad Sci U S A. 2007:104(29):12211–12216. 10.1073/pnas.0705186104 17609367 PMC1924578

[kiad318-B24] Navarro-Gochicoa MT , Camut S, Timmers AC, Niebel A, Herve C, Boutet E, Bono JJ, Imberty A, Cullimore JV. Characterization of four lectin-like receptor kinases expressed in roots of *Medicago truncatula*. Structure, location, regulation of expression, and potential role in the symbiosis with *Sinorhizobium meliloti*. Plant Physiol. 2003:133(4):1893–1910. 10.1104/pp.103.027680 14630957 PMC300742

[kiad318-B25] Okazaki S , Kaneko T, Sato S, Saeki K. Hijacking of leguminous nodulation signaling by the rhizobial type III secretion system. Proc Natl Acad Sci U S A. 2013:110(42):17131–17136. 10.1073/pnas.1302360110 24082124 PMC3801068

[kiad318-B26] Okazaki S , Tittabutr P, Teulet A, Thouin J, Fardoux J, Chaintreuil C, Gully D, Arrighi JF, Furuta N, Miwa H, et al Rhizobium-legume symbiosis in the absence of Nod factors: two possible scenarios with or without the T3SS. ISME J. 2016:10(1):64–74. 10.1038/ismej.2015.103 26161635 PMC4681849

[kiad318-B27] Oldroyd GED . Speak, friend, and enter: signalling systems that promote beneficial symbiotic associations in plants. Nat Rev Microbiol. 2013:11(4):252–263. 10.1038/nrmicro2990 23493145

[kiad318-B28] Oldroyd GED , Murray JD, Poole PS, Downie JA. The rules of engagement in the legume-rhizobial symbiosis. Annu Rev Genet. 2011:45(1):119–144. 10.1146/annurev-genet-110410-132549 21838550

[kiad318-B29] Perret X , Staehelin C, Broughton WJ. Molecular basis of symbiotic promiscuity. Microbiol Mol Biol Rev. 2000:64(1):180–201. 10.1128/MMBR.64.1.180-201.2000 10704479 PMC98991

[kiad318-B30] Pi L , Yin Z, Duan W, Wang N, Zhang Y, Wang J, Dou D. A G-type lectin receptor-like kinase regulates the perception of oomycete apoplastic expansin-like proteins. J Integr Plant Biol. 2022:64(1):183–201. 10.1111/jipb.13194 34825772

[kiad318-B31] Shamseldin A . Future outlook of transferring biological nitrogen fixation (BNF) to cereals and challenges to retard achieving this dream. Curr Microbiol. 2022:79(6):171. 10.1007/s00284-022-02852-2 35476236

[kiad318-B32] Shiu SH , Bleecker AB. Plant receptor-like kinase gene family: diversity, function, and signaling. Sci STKE. 2001:2001(113):re22. 10.1126/stke.2001.113.re22 11752632

[kiad318-B33] Si Z , Guan N, Zhou Y, Mei L, Li Y, Li Y. A methionine sulfoxide reductase B is required for the establishment of *Astragalus sinicus-Mesorhizobium* symbiosis. Plant Cell Physiol. 2020:61(9):1631–1645. 10.1093/pcp/pcaa085 32618998

[kiad318-B34] Skorpil P , Saad MM, Boukli NM, Kobayashi H, Ares-Orpel F, Broughton WJ, Deakin WJ. NopP, a phosphorylated effector of *Rhizobium* sp. strain NGR234, is a major determinant of nodulation of the tropical legumes *Flemingia congesta* and *Tephrosia vogelii*. Mol Microbiol. 2005:57(5):1304–1317. 10.1111/j.1365-2958.2005.04768.x 16102002

[kiad318-B35] Sugawara M , Takahashi S, Umehara Y, Iwano H, Tsurumaru H, Odake H, Suzuki Y, Kondo H, Konno Y, Yamakawa T, et al Variation in bradyrhizobial NopP effector determines symbiotic incompatibility with Rj2-soybeans via effector-triggered immunity. Nat Commun. 2018:9(1):3139–3151. 10.1038/s41467-018-05663-x 30087346 PMC6081438

[kiad318-B36] Tadege M , Wen J, He J, Tu H, Kwak Y, Eschstruth A, Cayrel A, Endre G, Zhao PX, Chabaud M, et al Large-scale insertional mutagenesis using the Tnt1 retrotransposon in the model legume *Medicago truncatula*. Plant J. 2008:54(2):335–347. 10.1111/j.1365-313X.2008.03418.x 18208518

[kiad318-B37] Takasaki T , Hatakeyama K, Suzuki G, Watanabe M, Isogai A, Hinata K. The S receptor kinase determines self-incompatibility in *Brassica stigma*. Nature. 2000:403(6772):913–916. 10.1038/35002628 10706292

[kiad318-B38] Teulet A , Busset N, Fardoux J, Gully D, Chaintreuil C, Cartieaux F, Jauneau A, Comorge V, Okazaki S, Kaneko T, et al The rhizobial type III effector ErnA confers the ability to form nodules in legumes. Proc Natl Acad Sci U S A. 2019:116(43):21758–21768. 10.1073/pnas.1904456116 31591240 PMC6815186

[kiad318-B39] Vaid N , Pandey PK, Tuteja N. Genome-wide analysis of lectin receptor-like kinase family from *Arabidopsis* and rice. Plant Mol Biol. 2012:80(4–5):365–388. 10.1007/s11103-012-9952-8 22936328

[kiad318-B40] Wang S , Hao B, Li J, Gu H, Peng J, Xie F, Zhao X, Frech C, Chen N, Ma B, et al Whole-genome sequencing of *Mesorhizobium huakuii* 7653R provides molecular insights into host specificity and symbiosis island dynamics. BMC Genomics. 2014:15(1):440–457. 10.1186/1471-2164-15-440 24906389 PMC4072884

[kiad318-B41] Wilson KJ , Sessitsch A, Corbo JC, Giller KE, Akkermans AD, Jefferson RA. beta-Glucuronidase (GUS) transposons for ecological and genetic studies of rhizobia and other gram-negative bacteria. Microbiology. 1995:141(7):1691–1705. 10.1099/13500872-141-7-1691 7551037

[kiad318-B42] Xiang QW , Bai J, Cai J, Huang QY, Wang Y, Liang Y, Zhong Z, Wagner C, Xie ZP, Staehelin C. NopD of *Bradyrhizobium* sp. XS1150 possesses SUMO protease activity. Front Microbiol. 2020:11:386–398. 10.3389/fmicb.2020.00386 32265858 PMC7098955

[kiad318-B43] Xin DW , Liao S, Xie ZP, Hann DR, Steinle L, Boller T, Staehelin C. Functional analysis of NopM, a novel E3 ubiquitin ligase (NEL) domain effector of *Rhizobium* sp. strain NGR234. PLoS Pathog. 2012:8(5):e1002707. 10.1371/journal.ppat.1002707 22615567 PMC3355095

[kiad318-B44] Xu N , Luo X, Wu W, Xing Y, Liang Y, Liu Y, Zou H, Wei HL, Liu J. A plant lectin receptor-like kinase phosphorylates the bacterial effector AvrPtoB to dampen its virulence in *Arabidopsis*. Mol Plant. 2020:13(10):1499–1512. 10.1016/j.molp.2020.09.016 32977056

[kiad318-B45] Yang S , Tang F, Gao M, Krishnan HB, Zhu H. R gene-controlled host specificity in the legume-rhizobia symbiosis. Proc Natl Acad Sci U S A. 2010:107(43):18735–18740. 10.1073/pnas.1011957107 20937853 PMC2973005

[kiad318-B46] Yasuda M , Miwa H, Masuda S, Takebayashi Y, Sakakibara H, Okazaki S. Effector-triggered immunity determines host genotype-specific incompatibility in legume-*Rhizobium* symbiosis. Plant Cell Physiol. 2016:57(8):1791–1800. 10.1093/pcp/pcw104 27373538

[kiad318-B48] Zhang B , Wang M, Sun Y, Zhao P, Liu C, Qing K, Hu X, Zhong Z, Cheng J, Wang H, et al *Glycine max* NNL1 restricts symbiotic compatibility with widely distributed bradyrhizobia via root hair infection. Nat Plants. 2021:7(1):73–86. 10.1038/s41477-020-00832-7 33452487

[kiad318-B47] Zhang L , Chen XJ, Lu HB, Xie ZP, Staehelin C. Functional analysis of the type 3 effector nodulation outer protein L (NopL) from *Rhizobium* sp. NGR234: symbiotic effects, phosphorylation, and interference with mitogen-activated protein kinase signaling. J Biol Chem. 2011:286(37):32178–32187. 10.1074/jbc.M111.265942 21775427 PMC3173237

[kiad318-B49] Zhou D , Li Y, Wang X, Xie F, Chen D, Ma B, Li Y. *Mesorhizobium huakuii* HtpG interaction with nsLTP AsE246 is required for symbiotic nitrogen fixation. Plant Physiol. 2019:180(1):509–528. 10.1104/pp.18.00336 30765481 PMC6501076

[kiad318-B50] Zhu H , Chen T, Zhu M, Fang Q, Kang H, Hong Z, Zhang Z. A novel ARID DNA-binding protein interacts with SymRK and is expressed during early nodule development in *Lotus japonicus*. Plant Physiol. 2008:148(1):337–347. 10.1104/pp.108.119164 18633121 PMC2528112

