# Supplementary material for: G-type receptor-like kinase AsNIP43 interacts with rhizobia effector nodulation outer protein P and is required for symbiosis
Source: Plant Physiol. 2023 Jul 11;193(2):1527–46. doi: 10.1093/plphys/kiad318 (PMC10517198; doi:10.1093/plphys/kiad318)
Supplement: kiad318_Supplementary_Data [file kiad318_supplementary_data.zip › PP2022RA00605DR2_Supplemental_Data.pdf]

Supplemental Methods, Figures and Tables

Supplemental Methods S1 Supplemental materials and methods describing secretion assay, construction and screening of *Astragulas sinicus* yeast two-hybrid cDNA library, observation and quantification of early infection events, RT-qPCR, and RNA-seq.

Supplemental Figure S1 T3SS and effector NopP of *Mesorhizobium huakuii* 7653R.

Supplemental Figure S2 Phylogenetic analysis of NopP homologs.

Supplemental Figure S3 Effects of 7653R*nopP* on rhizobial growth under free-living condition.

Supplemental Figure S4 Expression levels of the *nopP* in different mutants under free-living condition.

Supplemental Figure S5 KEGG pathway enrichment analysis of DEGs in host inoculated with 7653RΔ*nopP*.

Supplemental Figure S6 Sequence alignment of AsNIP43 and homologs from other legume plants.

Supplemental Figure S7 Phylogenetic analysis of AsNIP43 homologs.

Supplemental Figure S8 Expression levels of the *AsNIP43* and *nopP* genes at different time points after inoculation.

Supplemental Figure S9 Venn diagrams showing the comparative analysis KEGG pathways in RNA-seq of *A. sinicus* roots inoculated with 7653R*ΔnopP* and AsNIP43 RNAi hairy roots, and *A. sinicus* roots inoculated with 7653R*ΔnopP* and *Mtrlk* mutant roots.

Supplemental Figure S10 Expression profiles of *MtRLK* gene in the database of Bio-Analytic Resource.

Supplemental Figure S11 Model for the regulation of a LecRLK AsNIP43 on the symbiosis in *A. sinicus*.

Supplemental Table S1 The strains and plasmids used in this study.

Supplemental Table S2 Primers used in this study.

Supplemental Table S3 Candidate interacting proteins from NopP bait screening of *A. sinicus* cDNA library.

Supplemental Table S4 Differentially expressed genes related to defense response in

CK vs AsNIP43-RNAi at 1dpi

Supplemental Table S5 The common KEGG pathways in RNA-seq of *A. sinicus* roots inoculated with 7653R*ΔnopP* and AsNIP43 RNAi hairy roots.

Supplemental Table S6 The common KEGG pathways in RNA-seq of *A. sinicus* roots inoculated with 7653R*ΔnopP* and *Mtrlk* mutant roots.

Supplemental Dataset S1 The FPKM of all genes in *A. sinicus* roots at 1 day and 6 day post inoculation with wild-type *M. huakuii* 7653R and *M. huakuii* 7653R-*ΔnopP*

Supplemental Dataset S2 The FPKM of all genes in *A. sinicus* of AsNIP43-RNAi hairy roots at 1 day and 7 day post inoculation with wild-type *M. huakuii* 7653R

Supplemental Dataset S3 The FPKM of all genes in *M. truncatula* wild-type (R108) and *Mtrlk* mutant roots at 1 day and 6 day post inoculation with wild-type *S. meliloti* 2011

Supplemental Methods S1 Supplemental materials and methods describing secretion assay, construction and screening of *Astragulas sinicus* yeast two-hybrid cDNA library, observation and quantification of early infection events, RT-qPCR, and RNA-seq.

Secretion assay

The *nopP* upstream promoter region and its N-terminal encoding sequence 400 bp was amplified by PCR with a MYC tag added to the C-terminal, and then inserted into the *Bam*H Ⅰ and *Hin*dⅢ sites of pBBR1MCS-5 expression vector (Sánchez et al., 2009). The resulting recombinant construct was conjugated into wild-type 7653R and T3SS mutant (*hrcJ* encoding a lipoprotein-associated outer membrane protein was deleted) (Viprey et al., 1998) strains by bi-parental mating, respectively. The resulting strains were grown at 28°C with flavonoids (a mixture of Apigenin, Luteolin and Naringenin, 1 μM each final concentration) induction for 48 h to OD_{600} approximately 0.8, respectively. Bacteria were removed by two consecutive centrifugations at 5000 rpm for 30 min. Subsequently, ammonium sulfate was added to the supernatant to a final saturation of 80% at 4°C. The pellets were collected by centrifugation (8000 rpm at 4°C for 30 min) and resuspended in 50 mM Tris-HCl. The samples were desalted overnight using a dialysis bag and concentrated using ultrafiltration at 4°C. The final products were separated by SDS-PAGE, followed by immunoblotting analysis with anti-MYC

antibody (Viprey et al., 1998; Liu et al., 2021).

Construction of *Astragulas sinicus* yeast two-hybrid cDNA library

For the construction of Y2H cDNA library, roots of *A. sinicus* were collected 2, 4, 6, 8, 10 and 12 d after inoculation with *M. huakuii* 7653R and mixed in equal amounts, the samples were frozen in liquid nitrogen and ground, the total RNA was extracted by TRIzol reagent. According to the manufacturer's instruction of the Matchmaker^{TM} Library Construction & Screening Kit (Clontech), the total RNA (2 μg) was reverse transcribed into single-stranded cDNA with primer CDS III. Double-stranded cDNA was further amplified by LD-PCR using Advantage 2 PCR Kit (Clontech). The purified double-stranded cDNA fragments and linearized pGADT7-Rec were co-transformed into yeast (*Saccharomyces cerevisiae*) AH109 cells. The transformants were selected on SD/-Leu auxotrophic medium plates. And the transformation efficiency was approximately 1.2×10^{6} CFU/3 μg pGADT7-Rec.

Yeast two-hybrid library screening and the choice for AsNIP43 encoding a receptor-like kinase

Using the *M. huakuii* 7653R T3SS effector nopP as the bait, we screened the yeast two-hybrid cDNA library of *A. sinicus*. 106 positive colonies were obtained and were detected by PCR with AD universal primers, empty vectors and some small fragments of AD vectors were excluded, and the AD vectors with complete ORFs or partial ORFs with complete domains were re-transformed into AH109 yeast strains. The grown bacteria were detected by filter paper photocopying, and the ability to grow on SD/-Leu-Trp-His-Ade+X-gal indicated that the target protein interacts with the bait. The cDNA fragments in these positive AD vectors were then sequenced and subjected to BlastP analysis. We ended up with 12 candidate target proteins (Supplemental Table S3), one of which is a receptor-like kinase, which we named AsNIP43 (NopP-Interacting-Protein 43).

In this project, we aimed to identify any candidate protein interacting with NopP and this target protein putatively involves in plant signaling pathway in host plant. On the other hand, we have read a lot of references related symbiotic signaling pathways and carried on rational thinking. We noticed that, for examples, nodulation factor receptor

NFR1/NFR5 and EPS receptor EPR3 belong to LysM receptor kinases. AtLORE that can sense LPS is a lectin-receptor kinase. Therefore AsNIP43, encoding a receptor-like kinase, aroused our strong research interest. Meanwhile, we found that AsNIP43 was highly expressed in the infected roots but not in mature nodules by RT-qPCR, suggesting that AsNIP43 may involves in the early symbiotic interactions between rhizobia and legumes. Therefore, we focused on the AsNIP43 and carried out systematic experimental research in this study.

Observation and quantification of early infection events

We labeled the wild-type 7653R and 7653R*ΔnopP* with GFP and obtained the two strains with constitutive GFP expression. The *A. sinicus* seedlings were inoculated with 7653R*ΔnopP*-GFP and 7653R-GFP. For transgenic plants, the AsNIP43-OE and RNAi plants were inoculated with 7653R-GFP. Early infection events, including root hair curling, infection threads, and nodular primordia, were observed and quantified at 3, 5, and 7 days post-inoculation using a fluorescence microscope (Olympus SZX-16) with the GFP channel and the fluorescence intensity is 70.

RT-qPCR

The roots and nodules were harvested separately at different time points after inoculation, and free-living M. huakuii 7653R cells cultured in TY liquid medium for 48 h were collected by centrifugation at 12000 rpm at 4°C for 2 min. The samples were frozen in liquid nitrogen and total RNA were extracted by TRIzol reagent according to the instruction manual (Life technologies, California, USA). First-strand cDNA was synthesized using RevertAid Reverse Transcriptase (Fermentas). Plant cDNA was synthesized using primer Oligo (dT) while bacteria cDNA was synthesized using random primers. RT-qPCR was performed using the SYBR Green Master (Roche). The data were normalized to *Asactin* and *rnpB* expression, and were analyzed by the $2^{-\Delta\Delta Ct}$ method. The experiment was carried out in three replicates.

RNA-seq

For *A. sinicus* RNAseq analysis, roots of *A. sinicus* were collected 1 and 6 d post inoculation with *M. huakuii* 7653R or *ΔnopP* mutant. The harvested plant samples were frozen in liquid nitrogen and ground, and total RNA were extracted by TRIzol reagent

according to the instruction manual (Life technologies, California, USA). The integrity and concentration of total RNA were checked by an Agilent 2100 Bioanalyzer (Agilent Technologies, Inc., Santa Clara, CA, USA). The mRNA was enriched by NEBNext Poly (A) mRNA Magnetic Isolation Modele (NEB, E7490). The RNA-seq cDNA libraries were constructed using NEBNext Ultra RNA Library Prep Kit for Illumina (NEB, E7530) and NEBNext Multiplex Oligos for Illumina (NEB, E7500) according to the manufacturer's instructions. The prepared cDNA libraries were sequenced on an Illumina HiSeq 6000 platform. Raw RNA-seq data in fastq format were processed to remove the adapters and low quality sequences. Due to the lack of reference genome information available at the time, the generated sequences were subjected to *De novo* assembly by Trinity v 2.8.5. Use DIAMOND software to align the resultant unigenes with NCBI non-redundant (NR) protein, Swiss-Prot, COG, KOG, eggNOG4.5, KEGG database. The genes were identified with an E-value 10^{-5} against sequences in the database. FPKM values (fragments per kilobase of exon per million fragments mapped) were used to estimate gene expression levels (Trapnell et al., 2010). Differentially expressed genes (DEGs) were determined with false discovery rate (FDR) < 0.05 and $\log_2$(fold change, FC) >1 as the threshold using EdgeR v.3.18.1 (Robinson et al., 2010). GO enrichment analysis of all DEGs was performed using blast2GO (Götz et al., 2008). For *Medicago truncatula* R108 NF11649 RNAseq analysis, roots of *M. truncatula* R108 NF11649 were collected 1 and 6 d after inoculation with *Sinorhizobium meliloti* 2011. The harvested plant samples were frozen in liquid nitrogen and ground, and total RNA were extracted by TRIzol reagent (Invitrogen). The quality and concentration of total RNA were detected by an Agilent 2100 Bioanalyzer (Agilent Technologies, Santa Clara, CA, USA). The RNA-seq libraries were prepared with 1.5 μg of total RNA per sample by NEBNext Ultra RNA Library Prep Kit for Illumina (New England Biolabs, Ipswich, MA, USA) according to the manufacturer's instructions, and the attribute sequences of each sample were added with index codes. The index-coded samples' clustering was performed on a cBot Cluster Generation System using a HiSeq 4000 PE Cluster Kit (Illumina). The prepared libraries were sequenced on an Illumina HiSeq 4000 platform. The generated reads were aligned to the genome of *M. truncatula* Mt

4.0 (Tang et al., 2014). with default parameters by HISAT2. V.2.0.5 (Kim et al., 2015) and the transcripts read counts were generated by HTSEQ v.0.6.1 (Anders et al., 2015). FPKM was used to estimate gene expression levels. DEGs were determined with FDR < 0.05 and $\log_2(FC) > 1$ as the threshold using EdgeR v.3.18.1 (Trapnell et al., 2010). KEGG pathway analysis of all DEGs was used the KEGG Automatic Annotation Server (Moriya et al., 2007). Genes were identified with BLASTX with an E-value 10^{-5} against sequences of *Arabidopsis thaliana* TAIR10 (https://www.arabidopsis.org/) and *Lotus. japonicus* Lj 3.0 (http://www.kazusa.or.jp/lotus/).

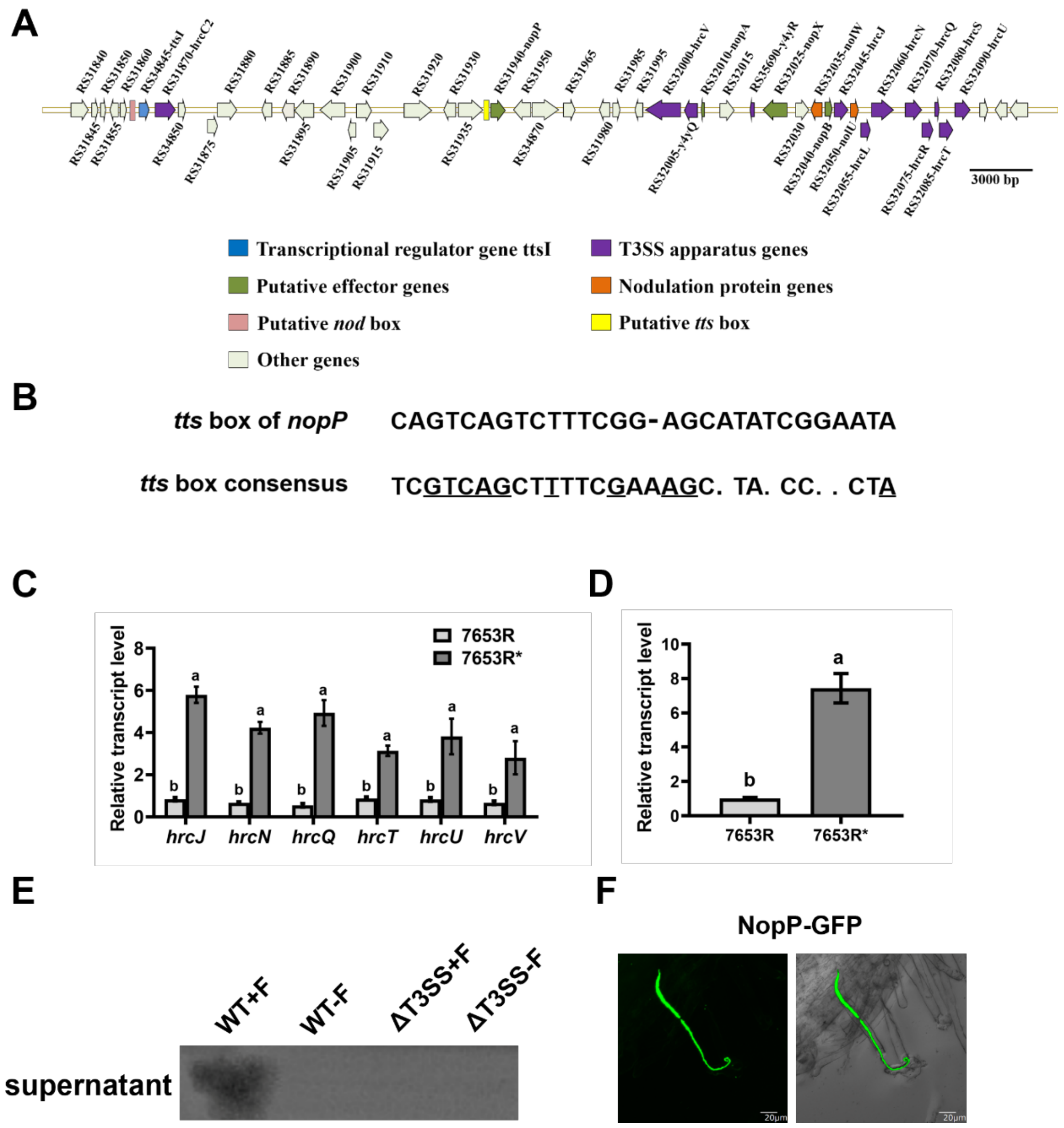

Supplemental Figure S1 T3SS and effector NopP of *Mesorhizobium huakuii* 7653R. A, A complete T3SS gene cluster was identified in the genome of *M. huakuii* 7653R. The gene cluster includes the transcriptional regulator gene *ttsI*, T3SS apparatus genes, and putative effector genes. Gene prediction was performed using Glimmer 3.0 software and GeneMark software was used for comparison. B, Alignment of the putative *tts* box of 7653R *nopP*. A putative *tts* box was found in the promoter region upstream of the ORF of the *nopP*. The underlined nucleotide sequence represents the consensus sequence of the putative *tts* box sequence of 7653R *nopP* and the conserved nucleotides of the *tts* box from various rhizobia. C-D. Expression levels of T3SS apparatus genes and *nopP* gene under flavonoids induction as detected by RT-qPCR.

rnpB was used as the reference gene. Left bars, 7653R without flavonoids induction and right bars, 7653R with flavonoids induction. Data are shown as averages ± SD of three biological replicates (*n*=3). Significant differences between the expression levels of each gene under different treatments according to Paired *t*-test are marked as a and b ($p < 0.05$). E, Secretion of MYC-fusion of NopP. Supernatant proteins were isolated from wild-type 7653R and *ΔT3SS* cultured with or without flavonoids. Proteins were separated by 12 % SDA-PAGE, followed by immunoblotting detection with anti-MYC antibody. F, NopP is expressed during the early stages of symbiosis. 7653R-NopP-GFP was inoculated to *A. sinicus* seedlings, GFP was observed at the infection thread. Scale bars, 20 μm.

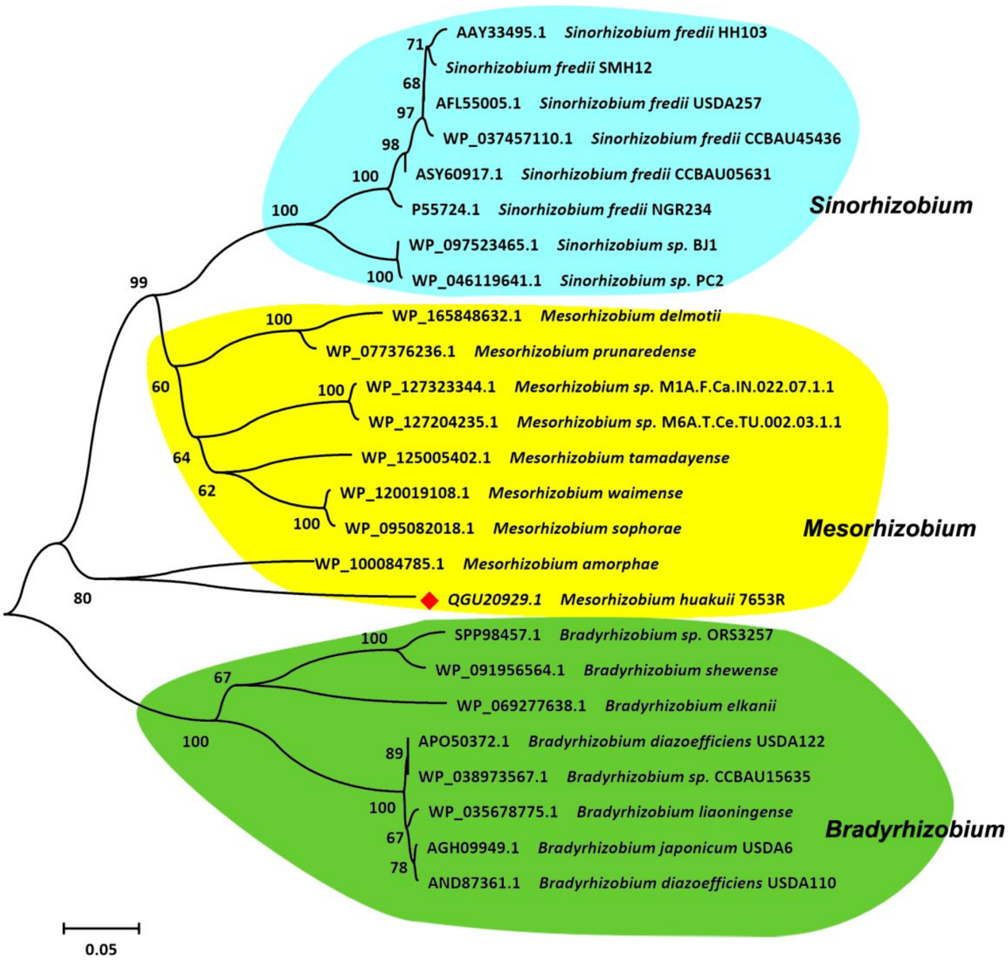

Supplemental Figure S2 Phylogenetic analysis of NopP homologs. The phylogenetic relationship was inferred by MEGA7.0 software using the Neighbor-Joining method. The evolutionary distance was computed using the p-distance method and presented in the number of amino acid differences per site. All ambiguous positions of the 25 amino acid sequences were eliminated, and a total of 274 positions were used to infer the tree. Different rhizobium genera are shaded with blue, yellow and green, respectively. The branch of *M. huakuii* 7653R NopP is marked with red color.

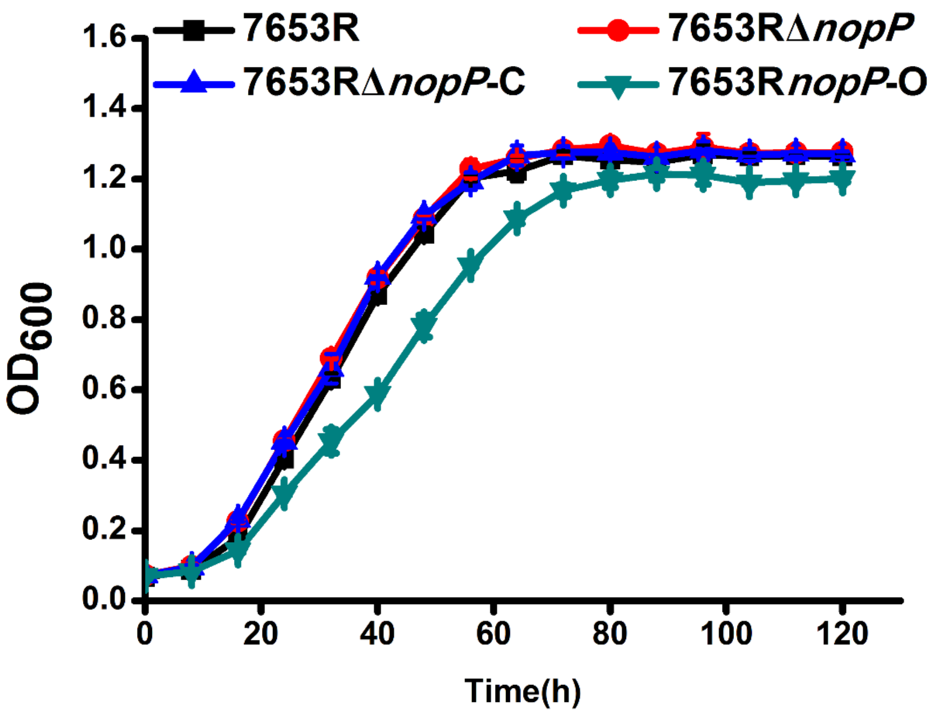

Supplemental Figure S3 Effects of 7653R *nopP* on rhizobial growth under free-living condition. Growth curves of wild-type 7653R, 7653RΔ*nopP*, 7653RΔ*nopP*-C and 7653R*nopP*-O. All of the strains were grown in liquid TY medium. The error bars represent the variant range of the data of three biological replicates.

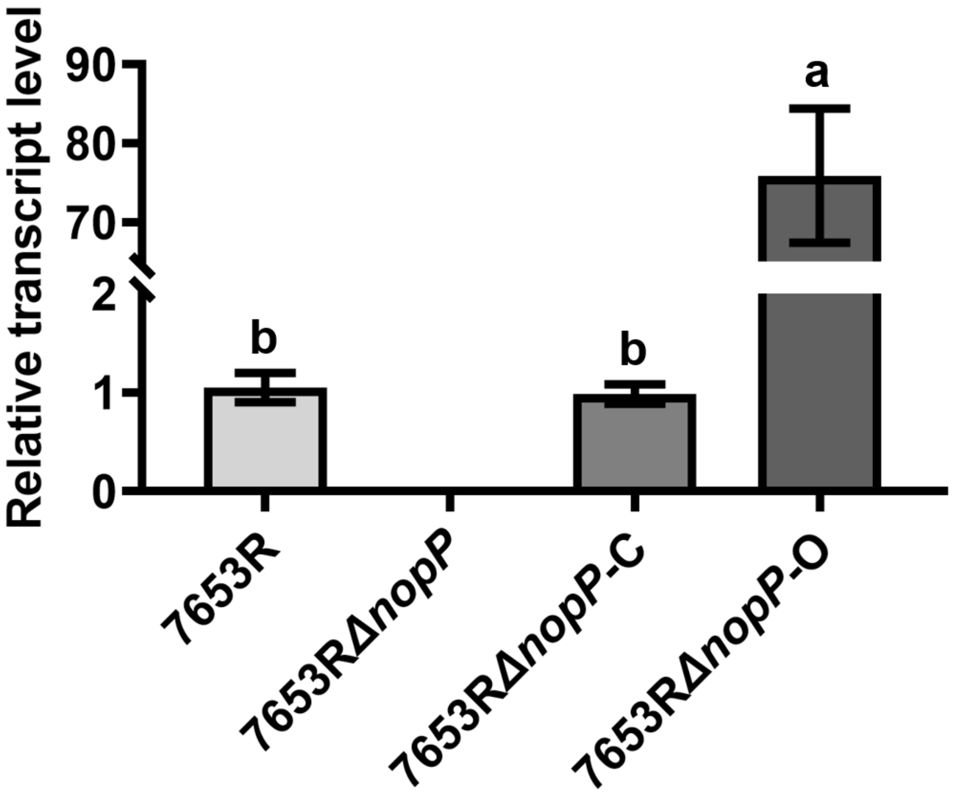

Supplemental Figure S4 Expression levels of the *nopP* in different mutants under free-living condition. Transcript levels of *nopP* in free-living 7653R, 7653R*ΔnopP*, 7653R*ΔnopP*-C and 7653R*ΔnopP*-O as detected by RT-qPCR. *rnpB* was used as the reference gene. Data are shown as averages ± SD of three biological replicates. Different letters of data represent significant differences according to one-way ANOVA multiple comparisons ($p < 0.05$).

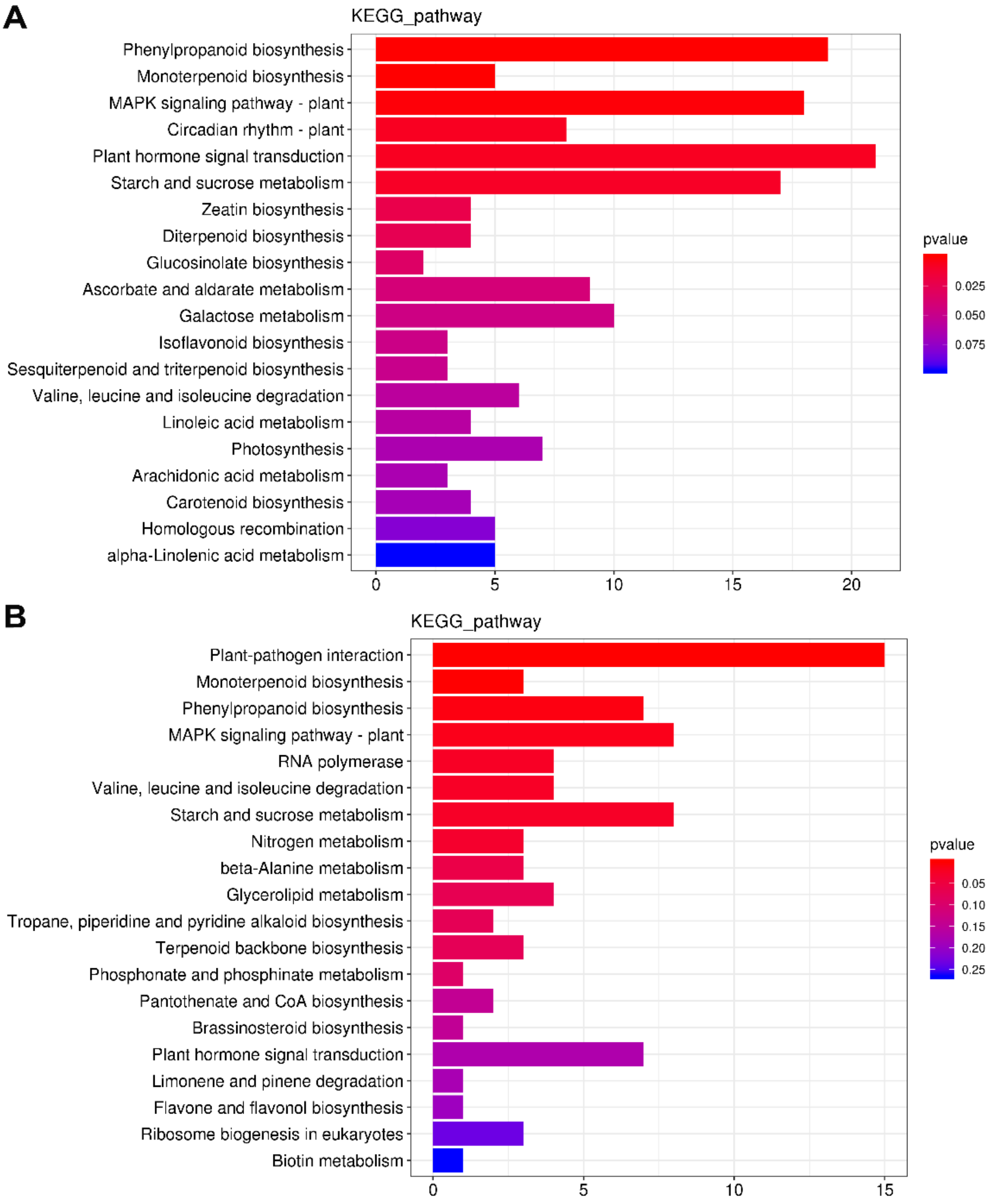

Supplemental Figure S5 KEGG pathway enrichment analysis of DEGs in host inoculated with 7653RΔ*nopP*. (A), KEGG pathway enrichment analysis of DEGs in WT-1 vs *nopP*-1 group; (B), KEGG pathway enrichment analysis of DEGs in WT-6 vs *nopP*-6 group.

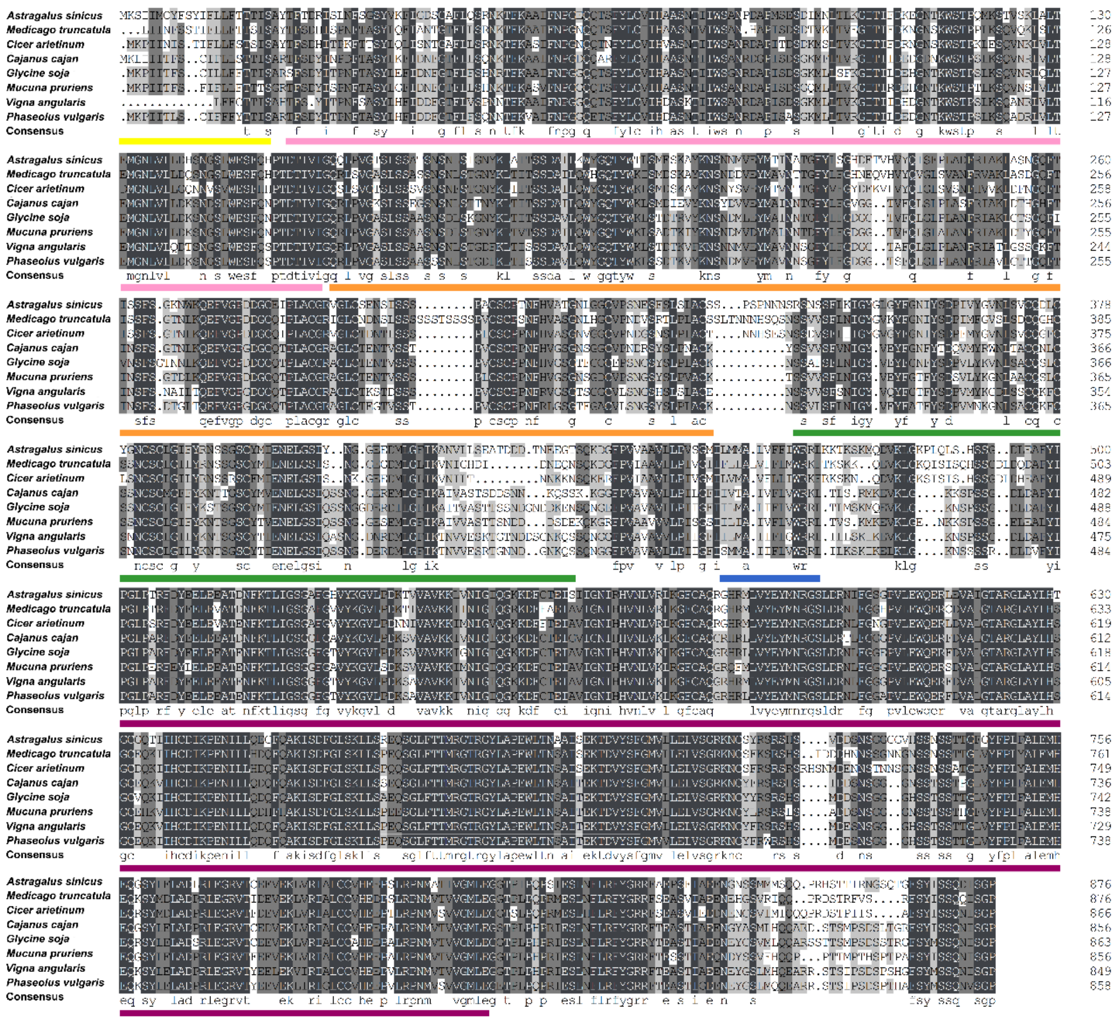

Supplemental Figure S6 Sequence alignment of AsNIP43 and homologs from other legume plants. Alignment of the AsNIP43 protein and homologs proteins. The predicted signal peptides are marked by yellow line. The N-terminal B-lectin, S-locus and PAN/APP domains are marked by pink, orange and green lines, respectively. The putative transmembrane domain is marked by blue line. The kinase domains are marked by purple line.

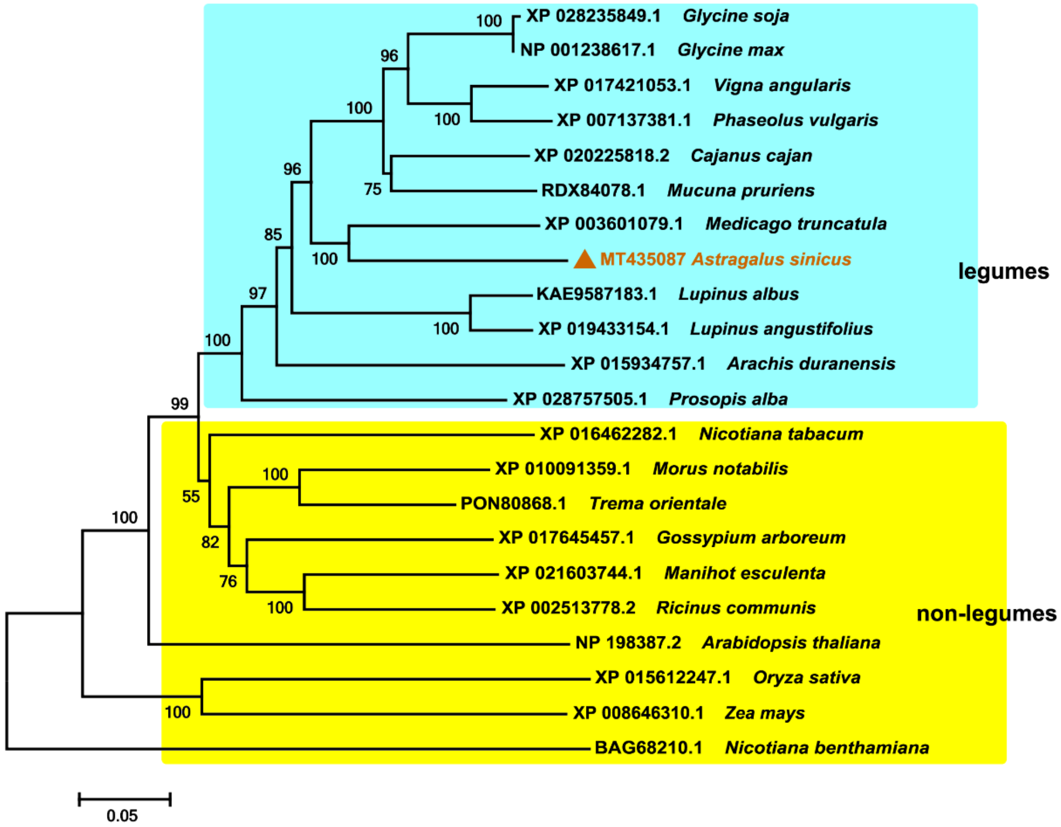

Supplemental Figure S7 Phylogenetic analysis of AsNIP43 homologs. The phylogenetic tree was generated by MEGA7.0 software with the Neighbour-Joining method. The bootstrap value (%) was set at 1000 replications and indicated at each branch node. The tree reveals the evolutionary relationship between AsNIP43 and homologs from different genera. Legumes (blue) and non-legumes (yellow) are shaded with different colors. The branch of AsNIP43 is marked with orange color. Bar, 0.05 sequence divergence.

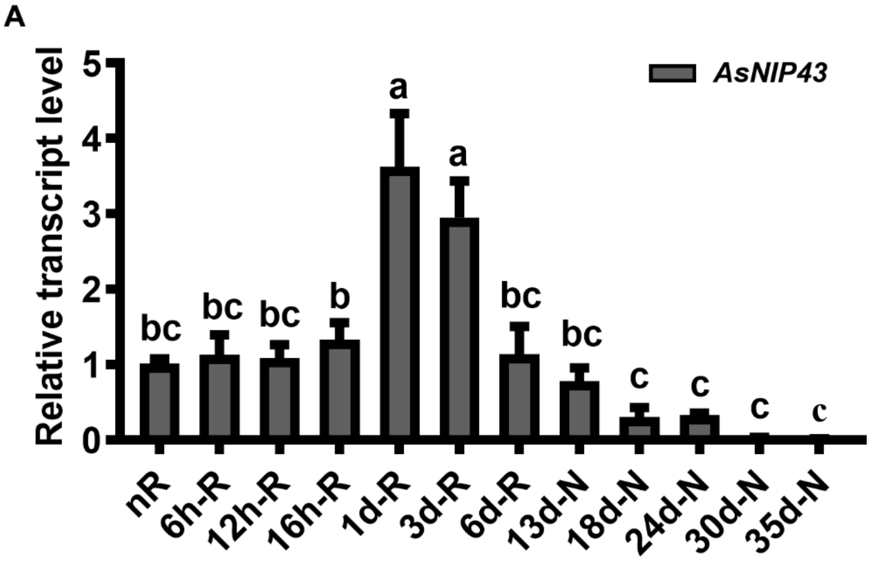

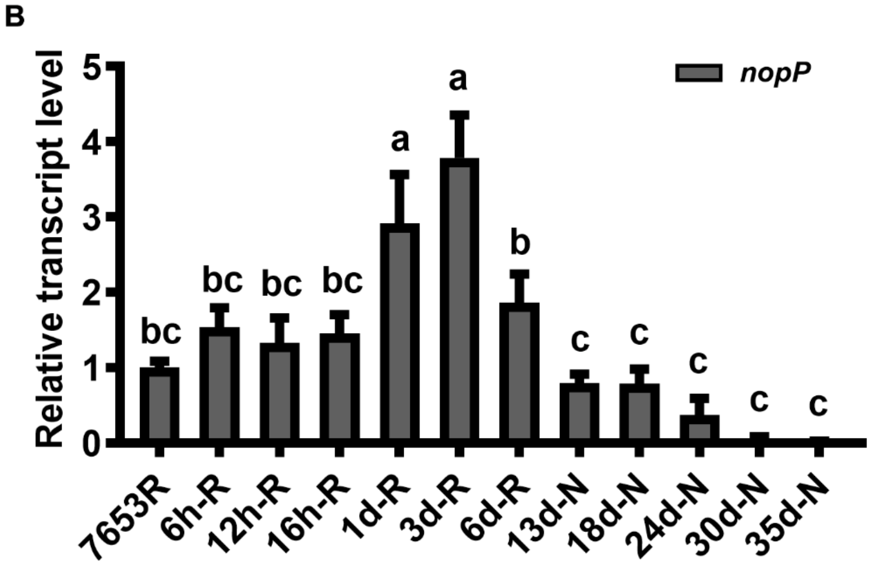

Supplemental Figure S8 Expression levels of the *AsNIP43* and *nopP* genes at different time points after inoculation. (A), Transcript levels of *AsNIP43* in non-inoculated root, and roots (6 h, 12 h, 16 h, 1 d, 3 d, 6 d) and nodules (13 d, 18 d, 24 d, 30 d, 35 d) at different time points post inoculation as detected by RT-qPCR. The housekeeping gene *Asactin* was used as the endogenous gene. (B), Transcript levels of *nopP* in free-living 7653R, roots (6 h, 12 h, 16 h, 1 d, 3 d, 6 d) and nodules (13 d, 18 d, 24 d, 30 d, 35 d) at different time points post inoculation as detected by RT-qPCR. *rnpB* was used as the reference gene. Data are shown as averages ± SD of three biological replicates. Different letters of data represent significant differences according to one-way ANOVA multiple comparisons ($p < 0.05$).

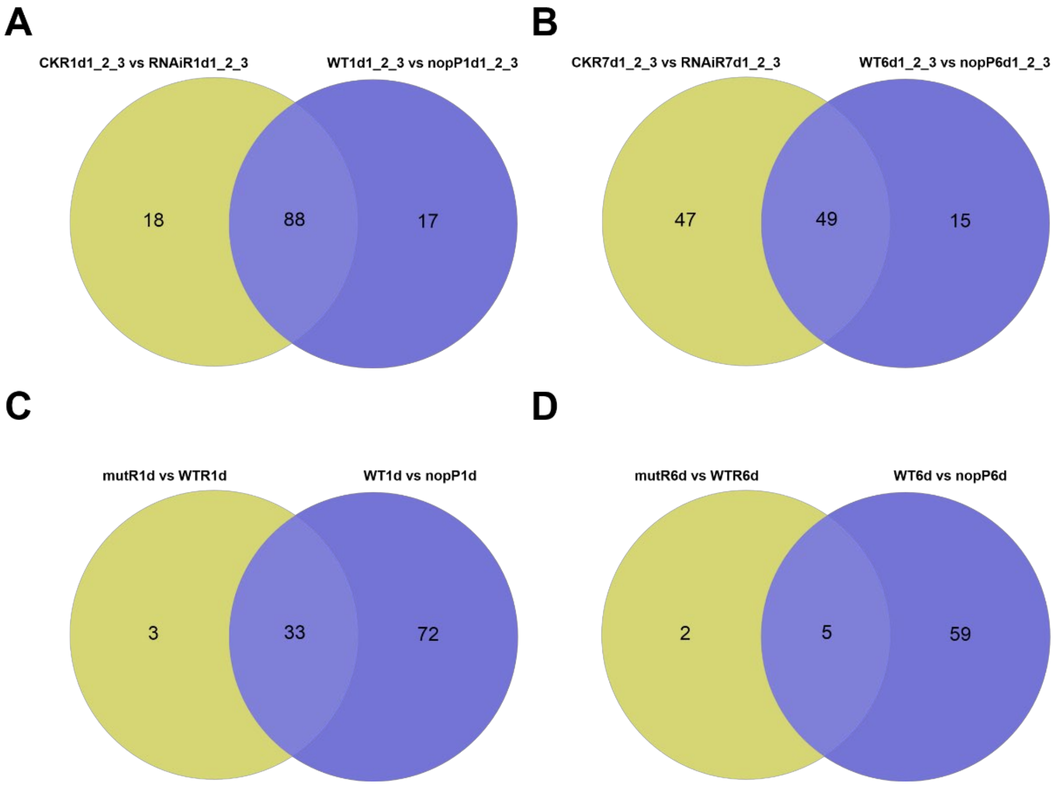

Supplemental Figure S9 Venn diagrams showing the comparative analysis KEGG pathways in 7653R*ΔnopP* inoculated *A. sinicus* roots and *Mtrlk* mutant roots, and 7653R*ΔnopP* inoculated *A. sinicus* roots and AsNIP43 RNAi roots. A, The comparative analysis KEGG pathways in AsNIP43-RNAi1d (yellow circle) and 7653R*ΔnopP*1d (blue circle); B, The comparative analysis KEGG pathways in AsNIP43-RNAi7d (yellow circle) and 7653R*ΔnopP*6d (blue circle); C, The comparative analysis KEGG pathways in mutR1d (yellow circle) and 7653R*ΔnopP*1d (blue circle); D, The comparative analysis KEGG pathways in mutR6d (yellow circle) and 7653R*ΔnopP*6d (blue circle).

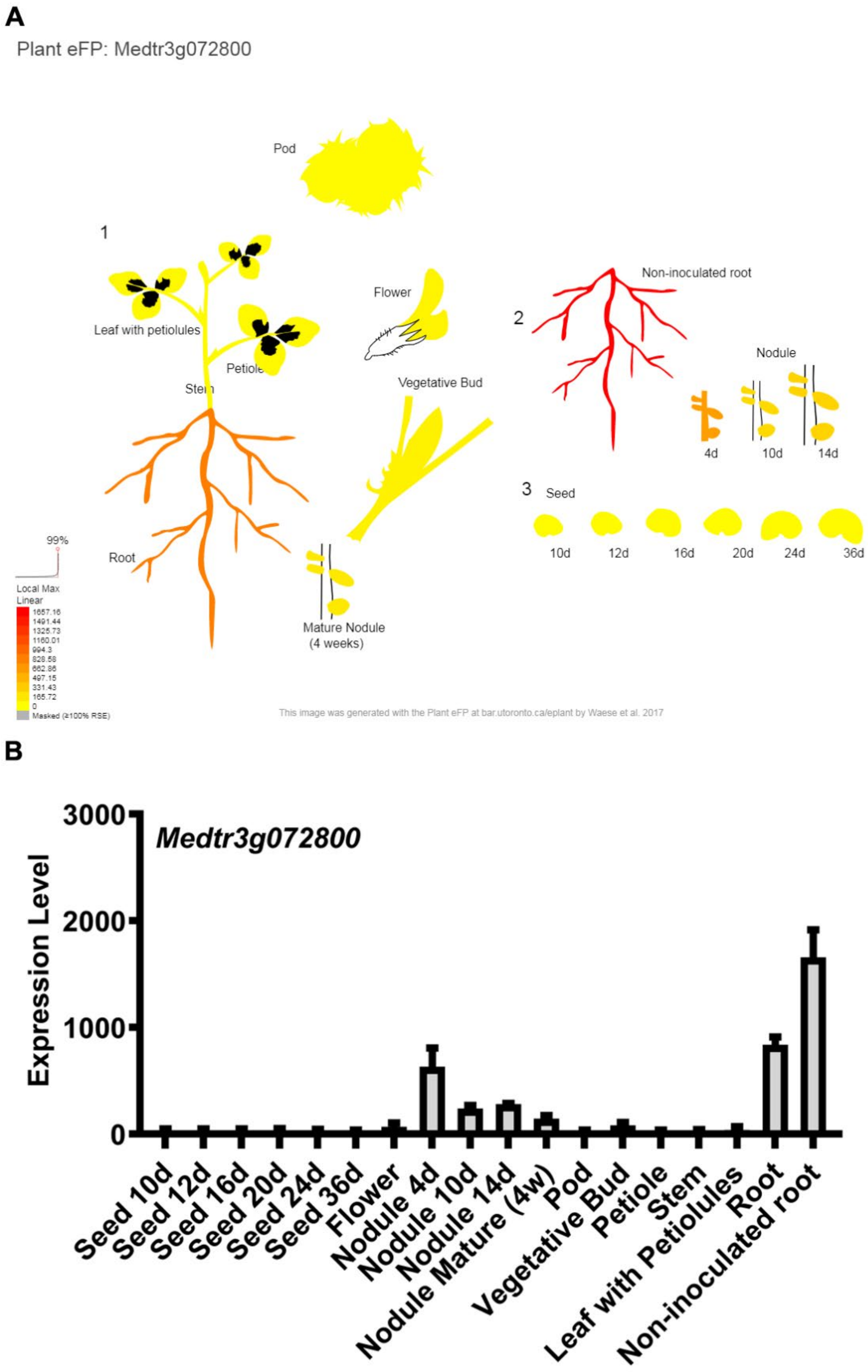

Supplemental Figure S10 Expression profiles of *MtRLK* gene in the database of Bio-Analytic Resource (http://bar.utoronto.ca/eplant_medicago/). *Medtr3g072800* corresponds to the *MtRLK* gene. A, The expression profile of *MtRLK* in different tissues and growth stages of *Medicago truncatula*; B, Quantitative expression profiles of *MtRLK* in different tissues and growth stages of *M. truncatula*. *MtRLK* transcripts are

abundant in roots, particularly in non-inoculated roots but quite low in mature nodules.

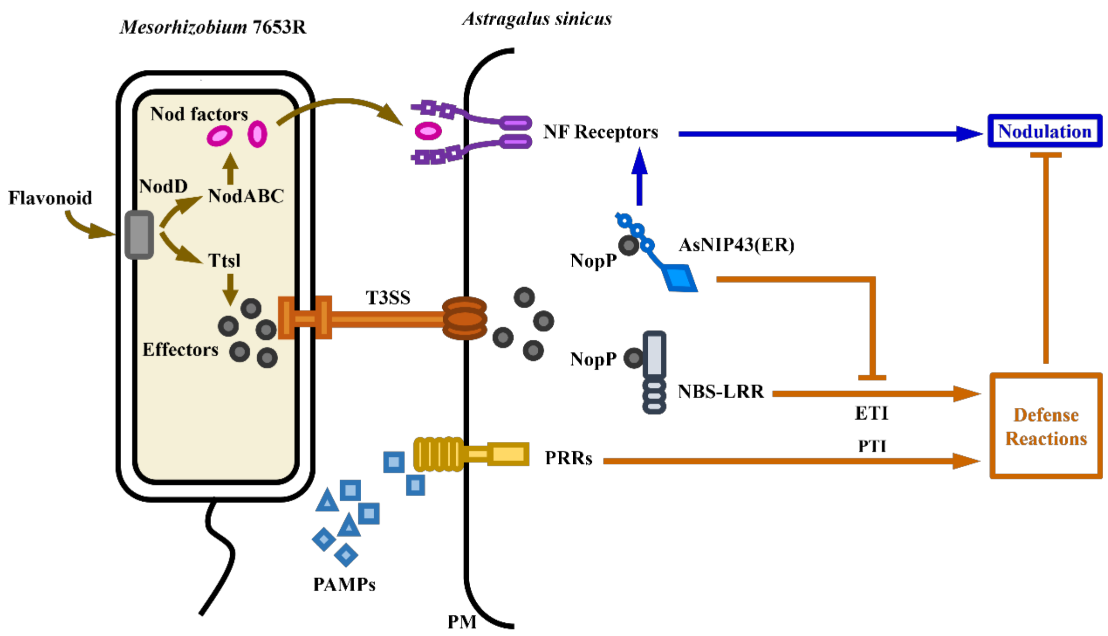

Supplemental Figure S11 Proposed model for the regulation of a LecRLK AsNIP43 on the symbiosis between *Mesorhizobium huakuii* 7653R and *A. sinicus*. The main ideas in the figure are as follows, NopP and AsNIP43 may work in the same pathway that related to symbiosis, *M. huakuii* 7653R NopP may negatively regulate the symbiotic relationship with the host plant *A. sinicus* by initiating defense responses via interacting with an unknown R protein (NBS-LRR) of *A. sinicus*. However, the interaction of a LecRLK AsNIP43 of *A. sinicus* with the 7653R effector NopP diverts the signaling pathway, and therefore weaken or block the usual ETI reaction mediated by an R protein. As a result, NopP-AsNIP43 interaction will enhance symbiotic nodulation. When the *nopP* of 7653R is deleted, the ETI response is attenuated and therefore enhance symbiosis. (PAMPs, Pathogen-associated molecular patterns; PM, Plasma membrane; NF, Nod factors; PRRs, Pattern recognition receptors; ER, Endoplasmic reticulum; NBS-LRR, Nucleotide-binding siteleucine-rich repeat; ETI, Effector-triggered immunity; PTI, PAMP-triggered immunity.)

Supplemental Table S1. The strains and plasmids used in this study

Strain or plasmid	Relevant characteristics	Reference or source
E. coli		
DH5α	*supE44 ΔlacU169(φ80lacZΔM15) hsdR17 recA1 endA1 gyrA96 thi-1 relA1*	TaKaRa
S17-1	*recA*, harbours the *tra* genes of plasmid RP4 in the chromosome; *proA*, *thi-1*	(Simon et al., 1983)
Rosetta(DE3)	F^-*ompThsdSB* (rB^- mB^-) *gal dcm*(DE3) $pRARE^2$ (Cam^r)	Invitrogen
M. huakuii		
7653R	Wild type, Nod^+, Fix^+, Str^r	This study
7653R-GFP	Wild type 7653R harboring the pMP2463 vector, Str^r, Gm^r	This study
ΔnopP	Deletion mutant of the *nopP*gene, Str^r	This study
ΔnopP-C	Complementation of *ΔnopP*by harboring the pBBR1MCS-5-*nopP*, Str^r, Gm^r	This study
nopP-O	Overexpression of 7653R by harboring the pBBR1MCS-5-*nopP*, Str^r, Gm^r	This study
7653R*ΔnopP* -GFP	*ΔnopP* harboring the pMP2463 vector, Str^r, Gm^r	This study
7653R*nopP*-GFP	Wild type 7653R harboring the pMP2463-*nopP*-GFP vector, Str^r, Gm^r	This study
S. meliloti		
2011	Wild type, Nod^+, Fix^+	This study
2011-GFP	Wild type 2011 harboring the pMP2463 vector, Nod^+, Fix^+, Gm^r	This study
S. cerevisiae		
AH109	*MATa,trp-901,leu2-3,ura3-52,his3-200,gal4Δ,gal80,LYS::GAL1*$_{UAS}$*-GAL1*$_{TATA}$*-HIS3,GAL2*$_{UAS}$*-GAL2*$_{TATA}$*-ADE2,URA3::MEL1*$_{USA}$*-MEL1*$_{TATA}$*-lacZ,MEL1*	Clontech
Y187	*MATa, ura3-52, ade2-101, trp1-901, his3-200, leu2-3, gal4Δ, gal80Δ, LYS::GAL1*$_{UAS}$*-GAL1*$_{TATA}$*-HIS3, GAL2*$_{UAS}$*-GAL2*$_{TATA}$*-ADE2, URA3::MEL1*$_{USA}$*-MEL1*$_{TATA}$*-lacZ, MEL1*	Clontech
A. tumefaciens		
GV3101(pMP90)	Host strain, Gm^r, Rif^r	(Koncz and Schell, 1986)
p19	Helper strain, Kan^r	(Voinnet et al., 2003)
A. rhizogenes		
K599	Host strain, Str^r	(Lei et al., 2014)
MSU440	Host strain, Str^r	(Limpens et al., 2004)
**Plasmids (bacteria)**		
pRG960	pRG930 containing the promoterless *gusA* with the start codon, Sp^r	(Van den Eede et al., 1992)
pCM184	Allelic exchange vector, Kan^r	(Marx and Lidstrom, 2002)
pCM157	The *cre* expression vector, Tc^r	(Marx and Lidstrom, 2002)
pBBR1MCS-5	Broad host-range expression vector, Gm^r	(Kovach et al., 1995)
pMP2463	pBBR1MCS5 derivate, Gm^r	(Stuurman et al., 2000)

Supplemental Table S1.Continued

Strain or plasmid	Relevant characteristics	Reference or source
pGADT7	Strep-tagged protein expression vector, Km^r	Clontech
pGBKT7	Broad host-range expression vector, Gm^r	Clontech
pET28a	His-tagged protein expression vector, Kan^r	Solarbio
pGEX-6P-1	GST-tagged protein expression vector, Ap^r	GE Healthcare
pMAL-c2x	MBP-tagged protein expression vector, Ap^r	(Walker et al., 2010)
**Plasmids (plant)**		
pXY104	Vector for BiFC analysis, Sp^r	(Wang et al., 2013)
pXY106	Vector for BiFC analysis, Sp^r	(Wang et al., 2013)
pCAMBIA1302-eGFP	Vector for subcellular localization and CoIP assay, Kan^r	(Wang et al., 2015)
pCAMBIA1302-DsRED	Vector for subcellular localization, Kan^r	(Wang et al., 2015)
pUB-GFP-3×HA	HA-tagged protein expression vector, Kan^r	Kindly proved by Prof.
DX2181G	Vector for promoter-GUS analysis, Kan^r	Zhongming Zhang,
pK7GWIWG2D(II)-RootRed	Vector for RNAi, Sp^r	(Karimi et al., 2002)
pDONR221	Vector for gateway, Kan^r	(Karimi et al., 2002)

Supplemental Table S2. Primers used in this study

Name	Sequence 5'→3'	Usage
nopP-Pro-F	ACGTTGTTGCCATTGCTGCAGTCCCACCTCCCAAGACTGC	Promoter-GUS analysis
nopP-Pro-R	TAAGGGACTGACCTACCCGGGCATCGCTCCTTGTCACACGAG	Promoter-GUS analysis
nopP-up-F	GCTGTACAATTGGTACCATGGTCAAAGCGCGACATTTTGAC	Homologous fragment
nopP-up-R	AAGTTATGCGGCCGCCATATGTAGTGCTACAATCCGGGCGT	Homologous fragment
nopP-down-F	GCTTATCGATACCGCGGGCCCCCGCCTTTCAACATACTCCG	Homologous fragment
nopP-down-F	TCCTCTAGTGAGCTCACCGGTGACAATCAGAAGACTGAAGCGAAG	Homologous fragment
nopP-Map-F	CGATCTGACCGTGGATGATG	Validation of *nopP* mutation
nopP-Map-R	TGATGTCGGCGATCCGACAG	Validation of *nopP* mutation
loxp-F	CAGGGTTATTGTCTCATGAGCGG	Validation of *nopP* mutation
loxp-R	CGACGCTCGAACGGGACTAC	Validation of *nopP* mutation
Kan-F	ATGAGCCATATTCAACGGGAAACGTCT	Cloning the ORF of Kan
Kan-R	TTAGAAAAACTCATCGAGCATCAAATGA	Cloning the ORF of Kan
nopP-756-F	TCAGGACAAGGCCAGCAGCG	Validation of *nopP* mutation
nopP-756-R	CTCAGAGTCATTGACGACACTG	Validation of *nopP* mutation
pBBR5-*nopP*-F	CGCTCTAGAACTAGTGGATCCTCCGCGATCGCCATATTG	Complemental fragment
pBBR5-*nopP*-R	GTCGACGGTATCGATAAGCTTTCACATGAAGTCGTCGTCGTCG	Complemental fragment
2463-*nopP*-F	CTATAGGGCGAATTGGAGCTCATGTCAATGGTGAACACAGGACG	GFP-fusion expression
2463-*nopP*-R	TAAAGCGGCCGCGACTCTAGACATGAAGTCGTCGTCGTCGG	GFP-fusion expression
nopP-BD-F	ATGGCCATGGAGGCCGAATTCATGTCAATGGTGAACACAGGACG	Yeast two-hybrid
nopP-BD-R	CCGCTGCAGGTCGACGGATCCTCACATGAAGTCGTCGTCGTCG	Yeast two-hybrid
N-B-AD-F	GCCATGGAGGCCAGTGAATTCTACACCTTTACTGATCGTATCAGTCTGA	Yeast two-hybrid
N-B-AD-R	CAGCTCGAGCTCGATGGATCCTCCAATAACAATTGTGTCAGTTGGA	Yeast two-hybrid
N-S-AD-R	CAGCTCGAGCTCGATGGATCCGGAAGGAGAACTACAAGCCAATG	Yeast two-hybrid
N-P-AD-R	CAGCTCGAGCTCGATGGATCCTTGAGAAGTTCCTTCTTCATTAGTGTC	Yeast two-hybrid
STKs-AD-F	GCCATGGAGGCCAGTGAATTCGTTAAGTTAGGAAAGCCATTGCAA	Yeast two-hybrid
STKs-AD-R	CAGCTCGAGCTCGATGGATCCTATTGGAGTTCCACCTTCCAACA	Yeast two-hybrid
nopP-nYFP-F	ATCGAGGACGCCGGCGGATCCATGTCAATGGTGAACACAGGACG	BiFC
nopP-nYFP-R	ACGAAAGCTCTGCAGGTCGACTCACATGAAGTCGTCGTCGTCG	BiFC
N-B-cYFP -F	ATTACAGGTACCCGGGGATCCATGTACACCTTTACTGATCGTATCAGTCT	BiFC
N-B-cYFP-R	CACGCTGCCACCGCCGTCGACTCCAATAACAATTGTGTCAGTTGGA	BiFC
N-S-cYFP-R	CACGCTGCCACCGCCGTCGACGGAAGGAGAACTACAAGCCAATG	BiFC
N-P-cYFP-R	CACGCTGCCACCGCCGTCGACTTGAGAAGTTCCTTCTTCATTAGTGTC	BiFC
AsNIP43-c-F	ATTACAGGTACCCGGGGATCCATGAAATCTATCATCATGTGTTATTTCTCG	BiFC
AsNIP43-c-R	CACGCTGCCACCGCCGTCGACTCTTGGCCCTGAGATATCTTGG	BiFC
His-*nopP*-F	GTGCCGCGCGGCAGCCATATGTCAATGGTGAACACAGGACGC	Protein expression
His-*nopP*-R	ACGGAGCTCGAATTCGGATCCTCACATGAAGTCGTCGTCGTCG	Protein expression

Supplemental Table S2. Continued

Name	Sequence 5'→3'	Usage
GST-*nopP*-F	TTCCAGGGGCCCCTGGGATCCTCAATGGTGAACACAGGACGC	Protein expression
GST-*nopP*-R	CTCGAGTCGACCCGGGAATTCTCACATGAAGTCGTCGTCGTCG	Protein expression
GST-PAN-F	TTCCAGGGGCCCCTGGGATCCTACACCTTTACTGATCGTATCAGTCTGA	Protein expression
GST-PAN-R	CTCGAGTCGACCCGGGAATTCCTATTGAGAAGTTCCTTCTTCATTAGTGT	Protein expression
MBP-PAN-F	GAGGGAAGGATTTCAGAATTCTACACCTTTACTGATCGTATCAGTCTGA	Protein expression
MBP-PAN-R	CAGGTCGACTCTAGAGGATCCCTATTGAGAAGTTCCTTCTTCATTAGTGT	Protein expression
AsNIP43-GFP-F	ACGGGGGACTCTTGACCATGGATGAAATCTATCATCATGTGTTATTTCTCG	Subcellular localization
AsNIP43-GFP-R	AAGTTCTTCTCCTTTACTAGTTCTTGGCCCTGAGATATCTTGG	Subcellular localization
nopP-GFP-F	ACGGGGGACTCTTGACCATGGATGTCAATGGTGAACACAGGACG	Subcellular localization
nopP-GFP-R	AAGTTCTTCTCCTTTACTAGTCATGAAGTCGTCGTCGTCGG	Subcellular localization
nopP-DsRED-F	ACGGGGGACTCTTGACCATGGATGTCAATGGTGAACACAGGACG	Subcellular localization
nopP-DsRED-R	CTTGGAAGACCTCATACTAGTCATGAAGTCGTCGTCGTCGG	Subcellular localization
nopP-HA-F	TTGATGTGATTACAGTCTAGAATGTCAATGGTGAACACAGGACG	Protein expression
nopP-HA-R	AACATCGTATGGGTAGGTACCCATGAAGTCGTCGTCGTCGG	protein expression
AsNIP43-Pro-F	TGATCTACAGCGCTGAAGCTTCCTCAAGAGGGGGAAAAATCGTATAG	Promoter-GUS analysis
AsNIP43-Pro-R	GGACTGACCACCCGGGGATCCCATGGTGTAATGATTTGTTGAAAAAC	Promoter-GUS analysis
MtRLK-Pro-F	TGATCTACAGCGCTGAAGCTTTATTTATATTAGTGATCGAAGGATATACATATTATT	Promoter-GUS analysis
MtRLK-Pro-R	GGACTGACCACCCGGGGATCCCATGGTGTGATAATTTGTTGAAAAATA	Promoter-GUS analysis
AsNIP43-OE-F	ACGCGTCGACTGCGGCAAGTCTGAAGT	Overexpression fragment
AsNIP43-OE-R	TCTAGACAACAAATCATTACACCATG	Overexpression fragment
AsNIP43-RNAi-SB-F	TCCCCCGGGCGTAGATTCGCTGAGCCTTCCT	RNAi fragment
AsNIP43-RNAi-SB-R	CGGGATCCACAGATGCAATCTTCCAGACCA	RNAi fragment
AsNIP43-RNAi-PS-F	AACTGCAGCGTAGATTCGCTGAGCCTTCCT	RNAi fragment
AsNIP43-RNAi-PS-R	GCACGCGTCGACACAGATGCAATCTTCCAG	RNAi fragment
AsNIP43-q-F	CTCCCTGATAAGACTGTGGTGGC	RT-qPCR
AsNIP43-q-R	GCACAAAACCCTTTCAGTCTCAC	RT-qPCR
Asactin-F	GTTCTTTTCCAGCCTTCTATGA	RT-qPCR
Asactin-R	ATGTTTCCGTACAGATCCTTTC	RT-qPCR
hrcJ-q-F	TGCCGCATAACGACCTTTTG	RT-qPCR
hrcJ-q-R	GCCCATCTGCGGACCCTTC	RT-qPCR

Supplemental Table S2. Continued

Name	Sequence 5'→3'	Usage
nopP-q-F	ACTAGCTGCCGAGGTTGCGC	RT-qPCR
nopP-q-R	CTTCTCCCGCCATCCATCGC	RT-qPCR
rnpB-q-F	AAGGCCGCAAGTGAGGAAAGTC	RT-qPCR
rnpB-q-R	GGTTTACCGTGCCGCTCCTGTTG	RT-qPCR
16s rRNA-F	TACGGGAGGCAGCAG	RT-qPCR
16s rRNA-R	ATTACCGCGGCTGCTGG	RT-qPCR
Tnt1-F	TCCTTGTTGGATTGGTAGCC	Validation of *rlk* mutation
Tnt1-R	CAGTGAACGAGCAGAACCTGTG	Validation of *rlk* mutation
rlk-1-F	TTCGTATCTTCAATTCATCGCTAACAC	Validation of *rlk* mutation
rlk-1-R	CACGACCCCGAGAGGAGCAAC	Validation of *rlk* mutation
rlk-2-F	ATGAAGATCCATCACTTAGGCC	Validation of *rlk* mutation
rlk-2-R	CTGAGATTTGTTGTGAAGATATG	Validation of *rlk* mutation
M13-F	CGCCAGGGTTTTCCCAGTCACGAC	Mutants screening
M13-R	AGCGGATAACAATTTCACACAGG	Mutants screening
hrcN-q-F	GGCGGATGATTGAACGACCC	RT-qPCR
hrcN-q-R	CAACGACGGCACGGCGAAGT	RT-qPCR
hrcQ-q-F	CGTGCCGGGGATGTTCTGTT	RT-qPCR
hrcQ-q-R	CAGCGGCCGCATTCAAAGAC	RT-qPCR
hrcT-q-F	CGCGCGTGATCCAGGTAACTC	RT-qPCR
hrcT-q-R	CCCCCACGGCGGAAGC	RT-qPCR
hrcU-q-F	AGCCGATCAAGCCCAGCCT	RT-qPCR
hrcU-q-R	CCCCGGCAGATACATCATGGTC	RT-qPCR
hrcV-q-F	AGCGAAAGGCGTGCAATCTC	RT-qPCR
hrcV-q-R	CGGCGCGATCAGCTTTGTC	RT-qPCR

Restriction enzyme sites are underlined.

Supplemental Table S3. Candidate interacting proteins from NopP bait screening of *A. sinicus* cDNA library

ID	Domain	Protein
A13	SGNH-hydrolase	Zinc finger protein
A32	SGNH-hydrolase	Zinc finger protein
A43	Receptor kinase	Receptor-like kinase protein
A45	TLP-PA	Thaumatin
B22	SERPIN	Serine protease inhibitor
B32	SelR	Chloroplast methionine sulfoxide reductase
B51	Glo-EDI-BRP	Lactate glutathione lyase
C22	Globin-like	Leghemoglobin
C53	HSP	Extensin-like protein
C55	Lipoyl	Dihydrolipoamide S-acetyltransferase
E2	SelR	Chloroplast methionine sulfoxide reductase
F36	SelR	Chloroplast methionine sulfoxide reductase

Supplemental Table S4 Differentially expressed genes related to defense response in CK vs AsNIP43-RNAi at 1dpi

ID	log2FC	regulated	description
c46174.graph_c1	1.023684	up	defense mechanisms
c53780.graph_c0	1.365599	up	disease resistance protein
c45636.graph_c0	1.210484	up	disease resistance protein
c42078.graph_c0	1.07322	up	WRKY transcription factor
c53851.graph_c1	1.089788	up	defense mechanisms
c56679.graph_c0	1.038155	up	disease resistance protein
c42511.graph_c0	2.389685	up	WRKY transcription factor
c48335.graph_c0	1.648505	up	disease resistance protein
c45047.graph_c0	2.218344	up	pathogenesis-related protein
c56462.graph_c0	1.238035	up	disease resistance protein
c55319.graph_c1	1.162308	up	disease resistance protein
c47892.graph_c0	2.077865	up	WRKY transcription factor
c54587.graph_c0	1.312919	up	defense mechanisms
c54274.graph_c0	1.106477	up	NBS-LRR resistance protein
c41328.graph_c0	1.59065	up	defense mechanisms
c49788.graph_c0	1.515776	up	disease resistance protein
c55483.graph_c0	1.250727	up	NBS-LRR resistance protein
c46324.graph_c0	1.423819	up	WRKY transcription factor
c43033.graph_c0	1.475871	up	defense mechanisms
c55756.graph_c0	1.269297	up	defense mechanisms
c46283.graph_c0	1.527811	up	WRKY transcription factor
c44424.graph_c0	1.777346	up	pathogenesis-related protein
c34916.graph_c0	1.257136	up	defensin-like protein
c49725.graph_c0	1.550284	up	disease resistance protein
c44452.graph_c0	1.078022	up	defense mechanisms

Supplemental Table S5 The common KEGG pathways in RNA-seq of *A. sinicus* roots inoculated with 7653R*ΔnopP* and AsNIP43 RNAi hairy roots.

time	ID	KEGG
1 dpi	ko00010	Glycolysis / Gluconeogenesis
	ko00020	Citrate cycle (TCA cycle)
	ko00030	Pentose phosphate pathway
	ko00040	Pentose and glucuronate interconversions
	ko00051	Fructose and mannose metabolism
	ko00052	Galactose metabolism
	ko00053	Ascorbate and aldarate metabolism
	ko00061	Fatty acid biosynthesis
	ko00062	Fatty acid elongation
	ko00071	Fatty acid degradation
	ko00073	Cutin, suberine and wax biosynthesis
	ko00100	Steroid biosynthesis
	ko00130	Ubiquinone and other terpenoid-quinone biosynthesis
	ko00190	Oxidative phosphorylation
	ko00195	Photosynthesis
	ko00196	Photosynthesis - antenna proteins
	ko00220	Arginine biosynthesis
	ko00230	Purine metabolism
	ko00250	Alanine, aspartate and glutamate metabolism
	ko00260	Glycine, serine and threonine metabolism
	ko00270	Cysteine and methionine metabolism
	ko00280	Valine, leucine and isoleucine degradation
	ko00290	Valine, leucine and isoleucine biosynthesis
	ko00310	Lysine degradation
	ko00330	Arginine and proline metabolism
	ko00340	Histidine metabolism
	ko00350	Tyrosine metabolism
	ko00360	Phenylalanine metabolism
	ko00380	Tryptophan metabolism
	ko00400	Phenylalanine, tyrosine and tryptophan biosynthesis
	ko00410	beta-Alanine metabolism
	ko00450	Selenocompound metabolism
	ko00460	Cyanoamino acid metabolism
	ko00480	Glutathione metabolism
	ko00500	Starch and sucrose metabolism
	ko00520	Amino sugar and nucleotide sugar metabolism
	ko00531	Glycosaminoglycan degradation
	ko00561	Glycerolipid metabolism
	ko00562	Inositol phosphate metabolism
	ko00564	Glycerophospholipid metabolism
	ko00565	Ether lipid metabolism
	ko00590	Arachidonic acid metabolism
	ko00591	Linoleic acid metabolism
	ko00592	alpha-Linolenic acid metabolism
	ko00600	Sphingolipid metabolism
	ko00620	Pyruvate metabolism
	ko00630	Glyoxylate and dicarboxylate metabolism
	ko00640	Propanoate metabolism
	ko00710	Carbon fixation in photosynthetic organisms
	ko00770	Pantothenate and CoA biosynthesis

	ko00780	Biotin metabolism
	ko00900	Terpenoid backbone biosynthesis
	ko00902	Monoterpenoid biosynthesis
	ko00906	Carotenoid biosynthesis
	ko00908	Zeatin biosynthesis
	ko00909	Sesquiterpenoid and triterpenoid biosynthesis
	ko00910	Nitrogen metabolism
	ko00940	Phenylpropanoid biosynthesis
	ko00941	Flavonoid biosynthesis
	ko00945	Stilbenoid, diarylheptanoid and gingerol biosynthesis
	ko00950	Isoquinoline alkaloid biosynthesis
	ko00960	Tropane, piperidine and pyridine alkaloid biosynthesis
	ko01200	Carbon metabolism
	ko01210	2-Oxocarboxylic acid metabolism 2
	ko01212	Fatty acid metabolism
	ko01230	Biosynthesis of amino acids
	ko02010	ABC transporters ABC
	ko03008	Ribosome biogenesis in eukaryotes
	ko03010	Ribosome
	ko03013	Nucleocytoplasmic transport
	ko03015	mRNA surveillance pathway mRNA
	ko03018	RNA degradation RNA
	ko03020	RNA polymerase RNA
	ko03030	DNA replication
	ko03040	Spliceosome
	ko03060	Protein export
	ko03420	Nucleotide excision repair
	ko03430	Mismatch repair
	ko03440	Homologous recombination
	ko04070	Phosphatidylinositol signaling system
	ko04075	Plant hormone signal transduction
	ko04120	Ubiquitin mediated proteolysis
	ko04141	Protein processing in endoplasmic reticulum
	ko04144	Endocytosis
	ko04145	Phagosome
	ko04146	Peroxisome
	ko04626	Plant-pathogen interaction
	ko04712	Circadian rhythm – plant
7 dpi	ko00010	Glycolysis / Gluconeogenesis
	ko00040	Pentose and glucuronate interconversions
	ko00052	Galactose metabolism
	ko00053	Ascorbate and aldarate metabolism
	ko00071	Fatty acid degradation
	ko00073	Cutin, suberine and wax biosynthesis
	ko00220	Arginine biosynthesis
	ko00250	Alanine, aspartate and glutamate metabolism
	ko00260	Glycine, serine and threonine metabolism Glycine, serine and threonine metabolism
	ko00270	Cysteine and methionine metabolism
	ko00280	Valine, leucine and isoleucine degradation
	ko00310	Lysine degradation
	ko00330	Arginine and proline metabolism

ko00340	Histidine metabolism
ko00350	Tyrosine metabolism
ko00360	Phenylalanine metabolism
ko00380	Tryptophan metabolism
ko00410	beta-Alanine metabolism
ko00480	Glutathione metabolism
ko00500	Starch and sucrose metabolism
ko00510	N-Glycan biosynthesis
ko00511	Other glycan degradation
ko00520	Amino sugar and nucleotide sugar metabolism
ko00561	Glycerolipid metabolism
ko00562	Inositol phosphate metabolism
ko00564	Glycerophospholipid metabolism
ko00565	Ether lipid metabolism
ko00591	Linoleic acid metabolism
ko00620	Pyruvate metabolism
ko00630	Glyoxylate and dicarboxylate metabolism
ko00770	Pantothenate and CoA biosynthesis
ko00904	Diterpenoid biosynthesis
ko00906	Carotenoid biosynthesis
ko00910	Nitrogen metabolism
ko00940	Phenylpropanoid biosynthesis
ko00941	Flavonoid biosynthesis
ko00943	Isoflavonoid biosynthesis
ko01230	Biosynthesis of amino acids
ko02010	ABC transporters ABC
ko03008	Ribosome biogenesis in eukaryotes
ko03018	RNA degradation
ko03040	Spliceosome
ko04075	Plant hormone signal transduction
ko04120	Ubiquitin mediated proteolysis
ko04141	Protein processing in endoplasmic reticulum
ko04144	Endocytosis
ko04145	Phagosome
ko04626	Plant-pathogen interaction
ko04712	Circadian rhythm – plant

Supplemental Table S6 The common KEGG pathways in RNA-seq of *A. sinicus* roots inoculated with 7653R*ΔnopP* and *Mtrlk* mutant roots.

time	ID	KEGG
1 dpi	ko00040	Pentose and glucuronate interconversions
	ko00052	Galactose metabolism
	ko00053	Ascorbate and aldarate metabolism
	ko00130	Ubiquinone and other terpenoid-quinone biosynthesis
	ko00190	Oxidative phosphorylation
	ko00270	Cysteine and methionine metabolism
	ko00280	Valine, leucine and isoleucine degradation
	ko00290	Valine, leucine and isoleucine biosynthesis
	ko00350	Tyrosine metabolism
	ko00360	Phenylalanine metabolism
	ko00400	Phenylalanine, tyrosine and tryptophan biosynthesis
	ko00450	Selenocompound metabolism
	ko00460	Cyanoamino acid metabolism
	ko00480	Glutathione metabolism
	ko00500	Starch and sucrose metabolism
	ko00520	Amino sugar and nucleotide sugar metabolism
	ko00770	Pantothenate and CoA biosynthesis
	ko00900	Terpenoid backbone biosynthesis
	ko00910	Nitrogen metabolism
	ko00940	Phenylpropanoid biosynthesis
	ko00941	Flavonoid biosynthesis
	ko00943	Isoflavonoid biosynthesis
	ko00944	Flavone and flavonol biosynthesis
	ko00950	Isoquinoline alkaloid biosynthesis
	ko00960	Tropane, piperidine and pyridine alkaloid biosynthesis
	ko01210	2-Oxocarboxylic acid metabolism 2
	ko01212	Fatty acid metabolism
	ko01230	Biosynthesis of amino acids
	ko03018	RNA degradation RNA
	ko04075	Plant hormone signal transduction
	ko04120	Ubiquitin mediated proteolysis
	ko04141	Protein processing in endoplasmic reticulum
	ko04626	Plant-pathogen interaction
6dpi	ko00052	Galactose metabolism
	ko00053	Ascorbate and aldarate metabolism
	ko00480	Glutathione metabolism
	ko00910	Nitrogen metabolism
	ko04626	Plant-pathogen interaction

Supplemental References

Anders S, Pyl PT, Huber W (2015) HTSeq--a Python framework to work with high-throughput sequencing data. Bioinformatics **31:** 166-169

Götz S, García-Gómez JM, Terol J, Williams TD, Nagaraj SH, Nueda MJ, Robles M, Talón M, Dopazo J, Conesa A (2008) High-throughput functional annotation and data mining with the Blast2GO suite. Nucleic Acids Res **36:** 3420-3435

Karimi M, Inzé D, Depicker A (2002) GATEWAY vectors for Agrobacterium-mediated plant transformation. Trends Plant Sci **7:** 193-195

Kim D, Langmead B, Salzberg SL (2015) HISAT: a fast spliced aligner with low memory requirements. Nat Methods **12:** 357-360

Koncz C, Schell J (1986) The promoter of TL-DNA gene5controls the tissue-specific expression of chimaeric genes carried by a novel type ofAgrobacteriumbinary vector. **204:** 383-396

Kovach ME, Elzer PH, Hill DS, Robertson GT, Farris MA, Roop RM, 2nd, Peterson KM (1995) Four new derivatives of the broad-host-range cloning vector pBBR1MCS, carrying different antibiotic-resistance cassettes. Gene **166:** 175-176

Lei L, Chen L, Shi X, Li Y, Wang J, Chen D, Xie F, Li Y (2014) A nodule-specific lipid transfer protein AsE246 participates in transport of plant-synthesized lipids to symbiosome membrane and is essential for nodule organogenesis in Chinese milk vetch. Plant Physiol **164:** 1045-1058

Limpens E, Ramos J, Franken C, Raz V, Compaan B, Franssen H, Bisseling T, Geurts R (2004) RNA interference in Agrobacterium rhizogenes-transformed roots of Arabidopsis and Medicago truncatula. J Exp Bot **55:** 983-992

Liu D, Luo Y, Zheng X, Wang X, Chou M, Wei G (2021) TRAPPC13 Is a Novel Target of Mesorhizobium amorphae Type III Secretion System Effector NopP. Mol Plant Microbe Interact **34:** 511-523

Marx CJ, Lidstrom ME (2002) Broad-host-range cre-lox system for antibiotic marker recycling in gram-negative bacteria. Biotechniques **33:** 1062-1067

Moriya Y, Itoh M, Okuda S, Yoshizawa AC, Kanehisa M (2007) KAAS: an automatic genome annotation and pathway reconstruction server. Nucleic Acids Res **35:** W182-185

Robinson MD, McCarthy DJ, Smyth GK (2010) edgeR: a Bioconductor package for differential expression analysis of digital gene expression data. Bioinformatics **26:** 139-140

Sánchez C, Iannino F, Deakin WJ, Ugalde RA, Lepek VC (2009) Characterization of the Mesorhizobium loti MAFF303099 type-three protein secretion system. Mol Plant Microbe Interact **22:** 519-528

Simon R, Priefer U, Puhler A (1983) A broad host mobilization system for in vivo genetic engineering: Transposon mutagenesis in Gram-negative bacteria. Bio/Technolgy **1:** 37-45

Stuurman N, Pacios Bras C, Schlaman HR, Wijfjes AH, Bloemberg G, Spaink HP (2000) Use of green fluorescent protein color variants expressed on stable broad-host-range vectors to visualize rhizobia interacting with plants. Mol Plant Microbe Interact **13:** 1163-1169

Tang H, Krishnakumar V, Bidwell S, Rosen B, Chan A, Zhou S, Gentzbittel L, Childs KL, Yandell M, Gundlach H, Mayer KF, Schwartz DC, Town CD (2014) An improved genome release (version Mt4.0) for the model legume Medicago truncatula. BMC Genomics **15:** 312

Trapnell C, Williams BA, Pertea G, Mortazavi A, Kwan G, van Baren MJ, Salzberg SL, Wold BJ, Pachter L (2010) Transcript assembly and quantification by RNA-Seq reveals unannotated transcripts and isoform switching during cell differentiation. Nat Biotechnol **28:** 511-515

Van den Eede G, Deblaere R, Goethals K, Van Montagu M, Holsters M (1992) Broad host range and promoter selection vectors for bacteria that interact with plants. Mol Plant Microbe Interact **5:** 228-234

Viprey V, Del Greco A, Golinowski W, Broughton WJ, Perret X (1998) Symbiotic implications of type III protein secretion machinery in Rhizobium. Mol Microbiol **28:** 1381-1389

Voinnet O, Rivas S, Mestre P, Baulcombe D (2003) An enhanced transient expression system in plants based on suppression of gene silencing by the p19 protein of tomato bushy stunt virus. Plant J **33:** 949-956

Walker IH, Hsieh PC, Riggs PD (2010) Mutations in maltose-binding protein that alter affinity and solubility properties. Appl Microbiol Biotechnol **88:** 187-197

Wang C, Zhu M, Duan L, Yu H, Chang X, Li L, Kang H, Feng Y, Zhu H, Hong Z, Zhang Z (2015) Lotus japonicus clathrin heavy Chain1 is associated with Rho-Like GTPase ROP6 and involved in nodule formation. Plant Physiol **167:** 1497-1510

Wang Y, Sun S, Zhu W, Jia K, Yang H, Wang X (2013) Strigolactone/MAX2-induced degradation of brassinosteroid transcriptional effector BES1 regulates shoot branching. Dev Cell **27:** 681-688
